# Mineralocorticoid receptor activation contributes to intestinal fibrosis through neutrophil gelatinase-associated lipocalin in preclinical models

**DOI:** 10.1038/s41467-025-61401-0

**Published:** 2025-07-09

**Authors:** Asma Amamou, Mathilde Leboutte, Jonathan Breton, David Ribet, Pierre-Alain Thiebaut, Christine Bôle-Feysot, Charlène Guérin, Kanhia Aublé, Elise Rebollo, Lise Ratel, Benjamin Bonnard, Alexis Goichon, Louison Leblond, Moutaz Aziz, Elodie Fermant, Frédéric Jaisser, Guillaume Savoye, Rachel Marion-Letellier

**Affiliations:** 1 Univ Rouen Normandie, INSERM, Normandie Univ, ADEN UMR 1073 Nutrition, Inflammation and Microbiota-Gut-Brain Axis, F-76000 Rouen, France; 2 Univ Rouen Normandie, Institute for Research and Innovation in Biomedicine (IRIB), F-76000 Rouen, France; 3 Department of Pathology, Charles Nicolle Hospital, Rouen University Hospital, Rouen, France; 4 Centre de Recherche des Cordeliers, Sorbonne Université, Inserm, Université Paris Cité, Team Diabetes, metabolic diseases and comorbidities, F-75006 Paris, France; 5 Université de Lorraine, INSERM Centre d’Investigations Cliniques-Plurithématique 1433, UMR 1116, CHRU de Nancy, French-Clinical Research Infrastructure Network (F-CRIN) INI-CRCT, Nancy, France; 6 Department of Gastroenterology, Rouen University Hospital, Rouen, France

**Keywords:** Gastrointestinal models, Physiology, Inflammatory bowel disease

## Abstract

Intestinal fibrosis is a common complication in inflammatory bowel diseases with no specific therapy. Because mineralocorticoid receptor antagonism prevented inflammation and fibrosis in extra-intestinal organs, we aimed to evaluate mineralocorticoid receptor antagonism in intestinal fibrosis. Here we show that pharmacological or smooth cell specific deletion mineralocorticoid receptor antagonism prevented colon fibrosis development in male mice. In vitro, spironolactone prevented fibroblast proliferation and endothelial-to-mesenchymal transition. Neutrophil gelatinase-associated lipocalin silencing suppressed aldosterone-induced fibrosis markers and blunted colon fibrosis in mice. Chromatin immunoprecipitation showed mineralocorticoid receptor antagonist inhibits mineralocorticoid receptor binding on the neutrophil gelatinase-associated lipocalin promoter in activated smooth muscle cells. In conclusion, mineralocorticoid receptor antagonism or smooth muscle mineralocorticoid receptor deletion reduced colon fibrosis through the modulation of the neutrophil gelatinase-associated lipocalin pathway. Mineralocorticoid receptor may represent a novel therapeutic target in intestinal fibrosis and may allow the re-positioning in the field of inflammatory bowel diseases of drugs already marketed.

## Abstract (web summary)

Here, the authors show that pharmacological and genetic mineralocorticoid receptor antagonism reduces intestinal fibrosis in mice and that NGAL is involved in mineralocorticoid receptor-mediated intestinal fibrogenesis.

## Introduction

The natural history of inflammatory bowel diseases (IBD) is frequently complicated by intestinal fibrosis and strictures formation. This is the case in more than 50% of Crohn’s disease (CD) and 5% of ulcerative colitis (UC) patients^1^. Intestinal fibrosis leads to surgery in CD and strictures frequently recur leading to repeated surgeries^2^. Intestinal fibrosis is a key therapeutic challenge in the management of IBD^3^. While control of inflammation by anti-inflammatory therapies such as anti-TNF have a modest effect to limit the development of intestinal fibrosis in IBD patients^4,5^, early treatment with anti-TNF agents can be effective in approximately a quarter of CD patients with symptomatic intestinal strictures^6^. It suggests the existence of inflammation-independent mechanisms contributing to intestinal fibrogenesis^3^.

Despite the increasing development of therapeutics such as biologics to control inflammation, therapies to prevent or inhibit intestinal fibrosis are very limited. The mineralocorticoid receptor (MR) is a key component of the renin-angiotensin-aldosterone system (RAAS) and it is the main regulator of renal sodium absorption and blood pressure. Targeting the MR is beneficial in cardiac and kidney fibrosis^7^. As the mechanisms that induce intestinal fibrosis are thought to be conserved between the intestine and extra-intestinal organs^2^, MR may represent a novel therapeutic target in intestinal fibrosis. Moreover, MR antagonists are already registered and usually safe drugs that are not contraindicated in IBD patients^8^.

All components of the RAAS are expressed within the gastrointestinal tract, including the MR that is highly expressed in the colon^9^ in both epithelial and smooth muscle cells (SMC)^10^. A recent clinical publication investigated the effects of RAAS inhibition on clinical outcomes in IBD patients^11^. The use of angiotensin-converting enzyme inhibitors and angiotensin receptor blockers led to fewer hospitalizations, operations, and corticosteroid use compared to matched controls^11^ but the effects of MR antagonism (MRA) have not been evaluated.

In the gut, serum neutrophil gelatinase-associated lipocalin (NGAL) is considered as a disease marker for active IBD^12^ and similarly, fecal NGAL is used as a pro-inflammatory marker in colitis models^13^. As we have identified NGAL as a novel MR-modulated target in the pro-inflammatory and pro-fibrotic processes induced by mineralocorticoids in heart, vessels and kidney^14^, we aimed to investigate its potential role in intestinal fibrosis mediated by MR signaling.

Here, we show that pharmacological and genetic MR antagonism reduces intestinal fibrosis in mice and that NGAL is involved in MR-mediated intestinal fibrogenesis.

## Results

### Colon MR expression and plasma aldosterone levels are increased in male mice with chronic DSS colitis

**Fig. 1:**
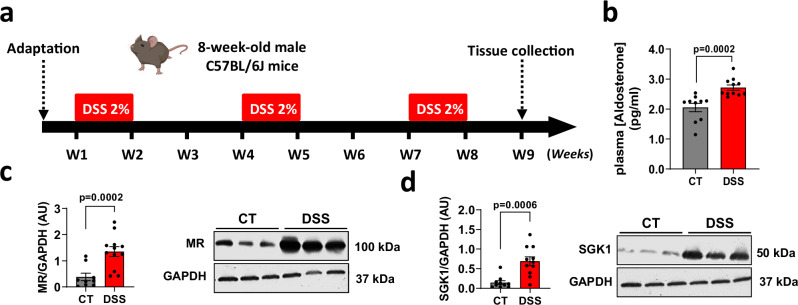
Dextran sulfate sodium (DSS)-induced chronic colitis and fibrosis upregulates plasma aldosterone and mineralocorticoid receptor (MR) activation. Male C57BL/6 J mice underwent 3 cycles of 2% DSS in their drinking water for 7 days, followed by 14 days of regular water (DSS; *n* = 11) whereas control mice received normal water (CT; *n* = 10). **a** Experimental design. **b** Plasma aldosterone level (*n* = 10 for CT, *n* = 11 for DSS; Two-sided Mann-Whitney test). **c, d** Representative western blot and relative protein expression of colon MR (**c**, *n* = 10 for CT, *n* = 11 for DSS; Two-sided Mann-Whitney test) and SGK1 (**d**, *n* = 9 for CT, *n* = 12 for DSS; Two-sided unpaired t-test with Welch’s correction). GAPDH is used as an internal control. Data are presented as mean values +/− SEM. Created in BioRender^67^.

Based on the hypothesis that targeting MR could be useful for inhibiting intestinal fibrosis, we first assessed the plasma level of the MR ligand, aldosterone, the level of colon MR expression, and the aldosterone-MR target, SGK1 in the chronic DSS colitis-induced intestinal fibrosis model (Fig. 1a). A significant increase in plasma aldosterone production (Fig. 1b), a higher colon MR protein level (Fig. 1c) and a higher colon SGK1 protein level (Fig. 1d) were observed in male mice with chronic DSS-induced colitis. These data indicate that MR signaling is increased in male mice with chronic DSS-induced colitis.

### Pharmacological MRA by spironolactone down-regulates intestinal fibrosis development in mice with chronic DSS colitis

**Fig. 2:**
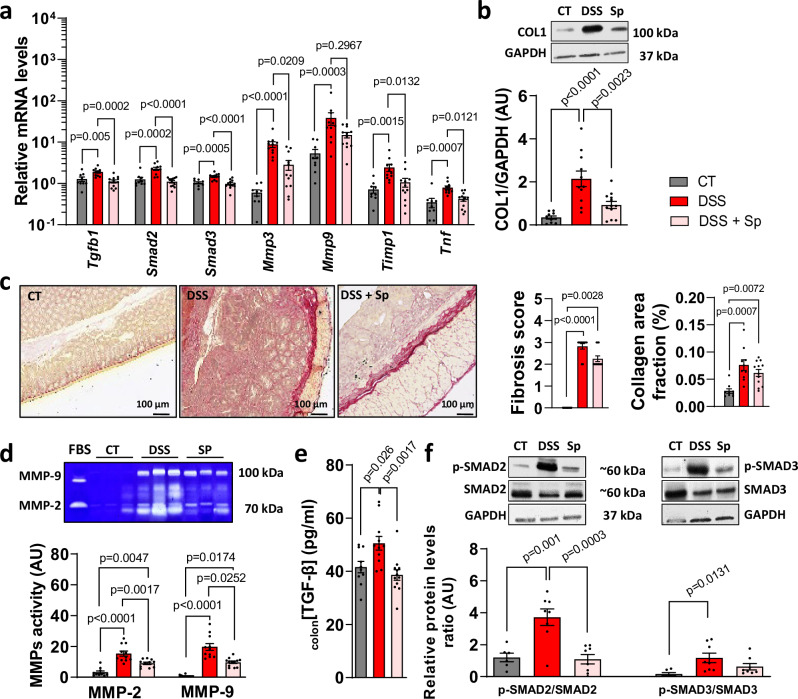
Mineralocorticoid receptor (MR) antagonism by spironolactone decreases intestinal fibrosis in mice with chronic colitis. **a–f** Pharmacological inhibition of MR by spironolactone in male mice with dextran sulfate sodium (DSS)-induced chronic colitis. Male C57BL/6 J mice underwent 3 cycles of 2% DSS in their drinking water for 7 days, followed by 14 days of regular water (DSS, *n* = 11) for both first cycles and 7 days for the third cycle whereas control mice received normal water (CT, *n* = 10). Mice were subjected to either a standard diet or diet supplemented with spironolactone (30 mg.kg^−1^; DSS+Sp, *n* = 12) throughout the experiment. (**a**) Colonic relative mRNA levels encoding for *Tgfb1, Smad2, Smad3, Mmp3, Mmp9, Timp1* and *Tnf* (*n* = 9 for CT, *n* = 11 for DSS, *n* = 12 for DSS+Sp; ordinary one way ANOVA with Tukey’s multiple comparisons test). **b** Representative western blot and relative protein expression of colon COL1 (*n* = 10 for CT, *n* = 11 for DSS, *n* = 12 for DSS+Sp; ordinary one way ANOVA with Tukey’s multiple comparisons test). **c** Sirius red stained colon sections, fibrosis score and collagen area fraction (*n* = 9 for CT, *n* = 11 for DSS, *n* = 12 for DSS+Sp; Kruskal-Wallis test with Dunn’s multiple comparisons test). **d** Representative gelatin zymography and quantification of colon MMP-9 (*n* = 10 for CT, *n* = 11 for DSS, *n* = 12 for DSS+Sp; Kruskal-Wallis test with Dunn’s multiple comparisons test) and −2 (*n* = 10 for CT, *n* = 11 for DSS, *n* = 11 for DSS+Sp; ordinary one way ANOVA with Tukey’s multiple comparisons test) activities. **e** Colonic TGF-β1 level (*n* = 10 for CT, *n* = 11 for DSS, *n* = 12 for DSS+Sp; ordinary one way ANOVA with Tukey’s multiple comparisons test). (**f**) Representative western blot and relative protein expression of phospho-SMAD 2/3 (p-SMAD2, p-SMAD3) and SMAD 2/3 (*n* = 6 for CT, *n* = 8 for DSS, *n* = 8 for DSS+Sp; ordinary one way ANOVA with Tukey’s multiple comparisons test for p-Smad2/Smad2, Kruskal-Wallis test with Dunn’s multiple comparisons test for p-Smad3/Smad3). Data are presented as mean values +/− SEM.

Next, we determined whether pharmacological MRA affects colitis and fibrosis by administering spironolactone to male mice exposed to 3 DSS cycles to induce chronic colitis with fibrosis (Supplementary Fig. 1a). Mice simultaneously treated with DSS and spironolactone exhibited significantly lower colon mRNA levels of fibrosis markers including *Tgfb1*, *Smad2*, *Smad3*, *Mmp3*, *Mmp9*, *Timp1*, *Tnf* than those treated with DSS alone (Fig. 2a). As increased collagen is a feature of intestinal fibrosis, we measured collagen levels in the colon by western blot and Red Sirius, which binds to all types of collagens. Spironolactone significantly reduced colon collagen 1 protein level (Fig. 2b) although it failed to significantly decrease histological fibrosis score and collagen area fraction (Fig. 2c). Because increased matrix metalloproteinases-2 and −9 (MMP) activities was associated with intestinal fibrosis, we analysed their activities by gelatin zymography. We found that spironolactone down-regulated colon MMP-2 and −9 activities in chronic DSS mice (Fig. 2d). Given that TGF-β/SMAD is critical in intestinal fibrosis, we then investigated whether spironolactone impact this signaling pathway. We observed that pharmacological MRA by spironolactone decreased colonic TGF-β1 production (Fig. 2e) and its associated SMAD2 signaling in chronic DSS-induced colitis mice (Fig. 2f).

In addition, spironolactone-treated mice exhibited a lower body weight loss than those treated with DSS alone (Supplementary Fig. 1b). This stabilization of body weight was associated with a reduced inflammation characterized by a decrease in chronic DSS-induced colon weight/length ratio (Supplementary Fig. 1c) and a lower inflammatory Nancy score (Supplementary Fig. 1d).

In contrast, spironolactone failed to significantly reduced colon IL-6 mRNA or protein levels or Mcp-1 mRNA levels, although it significantly reduced colon *Tnf* mRNA levels and pro-inflammatory cytokine IL-17 production (Supplementary Fig. 1e, f).

Lower colon MPO activity was observed in mice treated with spironolactone (Supplementary Fig. 1g). The p-NF-κB/NF-κB ratio in the colon was significantly upregulated in mice with chronic DSS, but not in spironolactone-treated mice with chronic DSS (Supplementary Fig. 1h). In addition, colon *Sgk1* mRNA and protein levels were lower in mice supplemented with spironolactone than those treated with DSS alone (Supplementary Fig. 1i, j). Together, these results indicate that MRA triggered by spironolactone attenuates colonic fibrosis and inflammation in mice with chronic DSS colitis.

### SMC-specific genetic deletion of the MR inhibits chronic DSS-induced intestinal fibrosis

Strictures are characterized by smooth muscle (SM) hypertrophy and hyperplasia and SMC represent the main cell component of the increased wall thickness in intestinal fibrosis^15^. Because MR is expressed in murine SMC (Smooth Muscle Transcriptome Browser^10^) and expansion of SM is involved in stricturing development, we examined the impact of cell-specific MR genetic deletion in SMC (illustrated in supplementary Fig. S2a) in mice with chronic DSS colitis. First, we observed that MR genetic deletion in SMC improved the survival rate in mice with chronic DSS-induced colitis (supplementary Fig. 2b) and reduced body weight loss (supplementary Fig. 2c). Female sm22-MR deleted mice exhibited lower inflammation with a reduced colon weight/length ratio (supplementary Fig. 2d) but did not show differences in Nancy index (supplementary Fig. 2e). Sm22-MR genetic deletion in female mice did not significantly impact colon MPO activity (supplementary Fig. 2f) or colon mRNA levels of *Tnf-α* (supplementary Fig. 2g) while it reduced colon mRNA levels encoding for *Il-*6 and *Mcp-1* (supplementary Fig. 2g). Sm22-MR deleted mice had a lower colon production of pro-inflammatory cytokines such as IL-6 or IL-17 (supplementary Fig. 2h), while colon NF-κB was not significantly modified (supplementary Fig. 2h). Female sm22-MR deleted mice exhibited lower fecal NGAL and calprotectin levels (supplementary Fig. 2j-k).

**Fig. 3:**
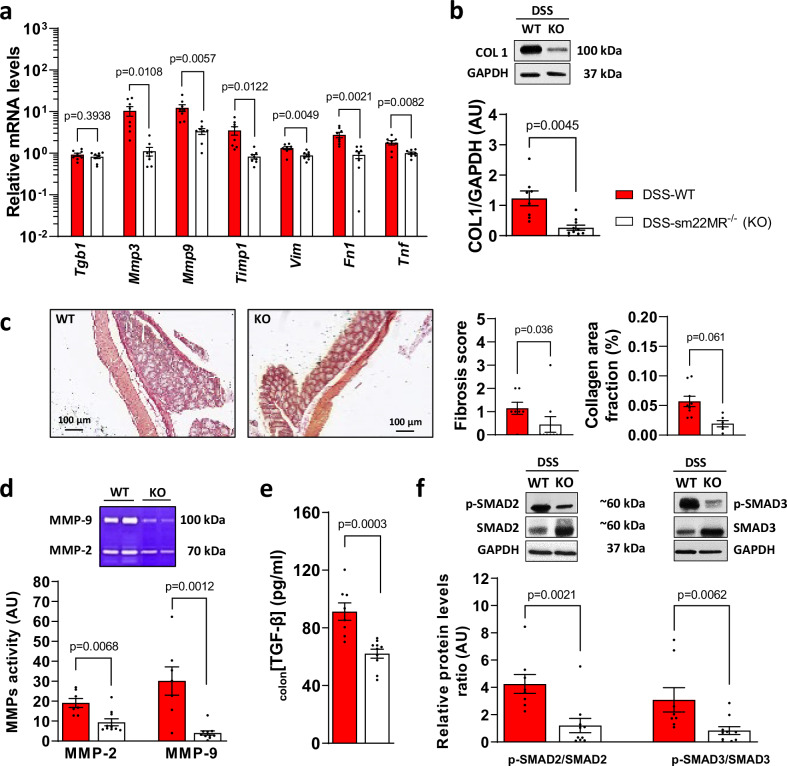
Genetic smooth muscle deletion of mineralocorticoid receptor (MR) decreases intestinal fibrosis in mice with chronic colitis. **a–f** Smooth muscle cell-specific inhibition of MR in female mice with dextran sulfate sodium (DSS)-induced chronic colitis. Female C57BL/6 J mice underwent 3 cycles of 2% DSS in their drinking water for 7 days, followed by 14 days of regular water for the both first cycles and 7 days for the third cycle (DSS-WT, *n* = 8; DSS-sm22MR^−/−^(KO), *n* = 10). **a** Colonic relative mRNA levels encoding for *Tgfb1, Mmp3, Mmp9, Timp1*, *Vim*, *Fn1* and *Tnf* (*n* = 8 per group; Two-sided unpaired t-test with Welch’s correction for *Mmp3, Mmp9, Timp1*, *Fn1* and *Tnf*; Two-sided unpaired t-test without Welch’s correction for *Tgfb1* and *Vim*). **b** Representative western blot and relative protein expression of colon COL1 (*n* = 8 for DSS-WT, *n* = 10 for DSS-sm22MR^−/−^; Two-sided unpaired t-test with Welch’s correction). **c** Sirius red colon sections, fibrosis score and collagen area fraction (*n* = 7 for DSS-WT, *n* = 9 for DSS-sm22MR^−/−^; Two-sided Mann-Whitney test for fibrosis score; Two-sided unpaired t-test without Welch’s correction for collagen area fraction). **d** Representative gelatin zymography and quantification of colon MMP-9 and MMP-2 activity (*n* = 7 for DSS-WT, *n* = 10 for DSS-sm22MR^−/−^; Two-sided Mann-Whitney test). **e** Colonic TGF-β1 level (*n* = 8 for DSS-WT, *n* = 10 for DSS-sm22MR^−/−^; Two-sided unpaired t-test without Welch’s correction). **f** Representative western blot and relative protein expression of phospho-SMAD 2/3 (p-SMAD2, p-SMAD3) and SMAD 2/3 (*n* = 8 for DSS-WT, *n* = 10 for DSS-sm22MR^−/−^; Two-sided Mann-Whitney test). Data are presented as mean values +/− SEM.

In line with the pharmalogical MR inhibition with spironolactone, female sm22-MR KO mice exhibited lower mRNA levels for *Mmp3*, *Mmp9*, *Timp1*, *Vim*, *Fn1* and *Tnf* (Fig. 3a), which were associated with lower colon collagen I (COL1) (Fig. 3b) compared to DSS-treated mice. Consistently, sm22-MR genetic deletion in female mice significantly reduced colon fibrosis score and collagen area fraction (Fig. 3c). We stained their colon with MR and α-SMA antibodies to assess by immunofluorescence its location. Images shown MR and α-SMA co-staining cells in the submucosa of DSS-WT mice contrary to Sm22-MR KO mice (supplementary Fig. 3).

Sm22-MR KO mice also exhibited lower colon MMP-2 and −9 activities (Fig. 3d). Colon TGF-β1 concentration (Fig. 3e) and its canonical SMAD signaling pathway (Fig. 3f) were lower in sm22-MR KO mice compared to WT mice. Sm22-MR deletion in male mice failed to significantly reduce fibrosis markers such as *Tgfb1*, *Mmp3*, *Mmp9*, *Timp1*, *Vim*, *Fn1* mRNA levels (supplementary Fig. 4a), collagen level (supplementary Fig. 4b), TGF-β1 level (supplementary Fig. 4c) and fibrosis score (supplementary Fig. 4d), although it reduced colon MMP-2 and −9 activities (supplementary Fig. 4e). In addition, sm22-MR KO male mice exhibited a reduced SMAD signaling pathway (supplementary Fig. 4f). NGAL mRNA level was reduced in male sm22-MR KO mice but did not reach statistical significance (p = 0.0531, supplementary Fig. 4g) and colon NGAL level was not significantly different among groups (supplementary Fig. 4h). In addition, sm22-MR deleted female mice also exhibited lower colon SGK1 protein and mRNA levels (supplementary Fig. 5a, b). These data suggest that MR genetic deletion in SMC prevented intestinal fibrosis in mice with DSS-induced chronic colitis.

### Pharmacological MRA by spironolactone did not impact intestinal fibrosis development in mice and rats with chronic TNBS colitis

To confirm these observations, we next determined whether MRA reduced fibrosis in a second commonly used model of intestinal fibrosis by using C57BL/6 J male mice with chronic TNBS-induced colitis^16^ (supplementary Fig. 6a).

Surprisingly, we did observe any significant effect of TNBS administration on body weight (supplementary Fig. 6b) compared to control mice. No difference in pro-inflammatory markers such as colon weight/length ratio (supplementary Fig. 6c) and fecal calprotectin levels (supplementary Fig. 6d) was observed between groups. Colonic mRNA levels of fibrotic parameters were not affected by chronic TNBS administration in male mice, except a reduction of colon *Col1a1* (supplementary Fig. 6e). Administration of spironolactone significantly reduced body weight compared to TNBS alone (supplementary Fig. 6b) and did not affect inflammation as shown by colon weight/length ratio (supplementary Fig. 6c) or fecal calprotectin levels (supplementary Fig. 6d). Most of extracellular matrix (ECM)-associated mRNA levels were not affected by spironolactone administration except an increase in *Col3* and *Tgfb1* mRNA levels compared to mice treated with TNBS alone (supplementary Fig. 6e).

As chronic TNBS administration in mice failed to induce fibrotic changes, we decided to use chronic TNBS-induced colitis in male rats (supplementary Fig. 7a). Male Sprague-Dawley rats were either fed with a standard diet or with a diet supplemented with spironolactone for 31 days. Chronic colitis was induced by weekly intra-rectal injection of 4 increasing doses of TNBS. TNBS administration reduced survival rate (supplementary Fig. 7b) and body weight compared to control rats (supplementary Fig. 7c). Fecal calprotectin levels were not different among groups (supplementary Fig. 7d) while TNBS challenges increased colon weight/length ratio in TNBS rats supplemented with spironolactone, but failed to reach statistical significance in rats treated with TNBS alone (supplementary Fig. 7e). TNBS-exposed rats with or without spironolactone supplementation had higher mRNA levels for ECM-associated proteins such as *Acta2*, *Col1a1* and *Vim* (supplementary Fig. 7f). These data indicate that spironolactone did not prevent inflammation and fibrosis in rats with chronic TNBS colitis.

### Aldosterone induces fibrosis-related genes expression in TGF-β1-activated Human colonic fibroblasts, and this effect is blunted by spironolactone treatment

**Fig. 4:**
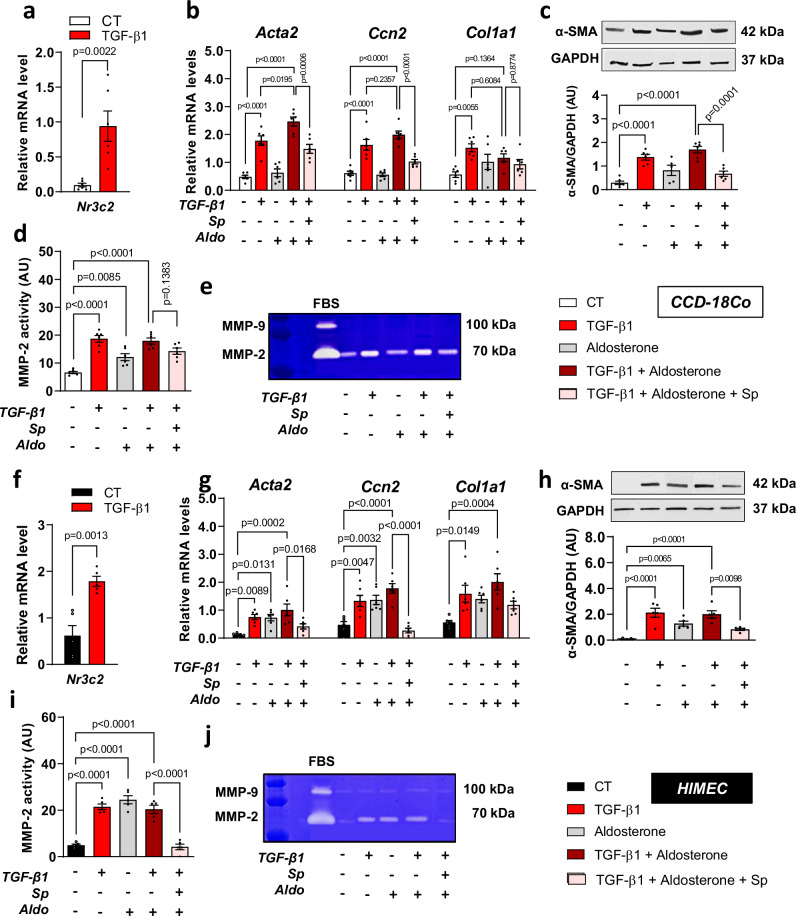
Mineralocorticoid receptor (MR) activation exacerbates extracellular matrix fibroblast synthesis and promotes endothelial mesenchymal transition. CCD-18Co (**a–e**) and HIMEC (**f–j**) cells were incubated with aldosterone (100 nM), spironolactone (10 µM) in response to TGF-β (10 ng/mL) for 24 h (*n* = 6 from independent experiments). (**a**) Relative mRNA levels of MR (*Nr3c2*) from CCD-18Co cell lysates (*n* = 6 per group; Two-sided Mann-Whitney test). **b** Relative mRNA levels of *Acta2, Ccn2,* and *Col1a1* from CCD-18Co cell lysates (*n* = 6 per group; ordinary one-way ANOVA with Tukey’s multiple comparisons test). **c** Relative protein expression of α-SMA from CCD-18Co cell lysates (*n* = 6 for CT, *n* = 6 for TGF-β1, *n* = 5 for Aldosterone, *n* = 6 for TGF-β1 + Aldosterone, *n* = 6 for TGF−β1 + Aldosterone + Sp; ordinary one-way ANOVA with Tukey’s multiple comparisons test). **d, e** Representative gelatine zymography and quantification of MMP-2 activity from CCD-18Co cell lysates (*n* = 6 per group; ordinary one-way ANOVA with Tukey’s multiple comparisons test). (**f**) Relative mRNA levels of MR (*Nr3c2*) from HIMEC cell lysates (*n* = 5 per group; Two-sided Mann-Whitney test). **g** Relative mRNA levels of *Acta2, Ccn2,* and *Col1a1* from HIMEC cell lysates (*n* = 6 per group; ordinary one-way ANOVA with Tukey’s multiple comparisons test). **h** Representative western blot and relative protein expression of α-SMA from HIMEC cell lysates (*n* = 5 per group; ordinary one-way ANOVA with Tukey’s multiple comparisons test). **i, j** Representative gelatin zymography and quantification of MMP-2 activity from HIMEC cell lysates (*n* = 5 per group; ordinary one-way ANOVA with Tukey’s multiple comparisons test). Data are presented as mean values +/− SEM.

Fibroblasts are key players in gastrointestinal fibrogenesis^17^. To investigate whether the effects of MRA were due to anti-fibrotic effects on fibroblasts, we investigated the effects of aldosterone and spironolactone on TGF-β1-induced fibroblast activation. First, aldosterone, a MR ligand, increased TGF-β1 mRNA levels in CCD-18Co cells (Supplementary Fig. 8a). In addition, either TGF-β1 or aldosterone incubation upregulated cell proliferation (Supplementary Fig. 8b). To elucidate the role of MR in colonic activated fibroblasts, we first studied the effect of TGF-β1 on MR (*Nr3c2*) mRNA expression in CCD-18Co cells and we found that TGF-β1 upregulated *Nr3c2* (Fig. 4a). Next, we evaluated the effect of the MR ligand aldosterone on fibrosis-associated markers in CCD-18Co cells. Aldosterone upregulates TGF-β1-induced *Acta2* mRNA (Fig. 4b) and protein (Fig. 4c) levels. TGF-β1 alone, or in combination with aldosterone, upregulates *Ccn2* and *Col1a1* mRNA levels, while aldosterone alone does not (Fig. 4b). Colon MMP-2 activity was upregulated by TGF-β1 and/or aldosterone (Fig. 4d, e). The MRA by spironolactone decreases aldosterone-induced *Acta2* mRNA and proteins levels, and CTGF mRNA levels (Fig. 4b, c).

### Aldosterone and TGF-β1 promote endothelial-mesenchymal transition

Studies have reported endothelial-mesenchymal transition (EndoMT) in microvessels of IBD mucosa and murine experimental colonic fibrosis^17^. As EndoMT is a major driver of intestinal fibrosis, we investigated the effects of MR activation on the activation of endothelial cells as other myofibroblasts progenitor sources. First, we studied the effect of aldosterone on *Tgfb1* mRNA levels, and we observed that aldosterone upregulated its expression (Supplementary Fig. 8c). TGF-β1 treatment increased MR (*Nr3c2*) mRNA levels in HIMEC (Fig. 4f). TGF-β1 treatment up-regulated *Acta2* mRNA and proteins levels (Fig. 4g, h), *Ccn2* mRNA and *Col1a1* mRNA levels in HIMEC (Fig. 4g). Aldosterone treatment up-regulated *Acta2* mRNA and protein levels in HIMEC (Fig. 4g, h) and also *Ccn2* mRNA levels (Fig. 4g) and MMP-2 activity (Fig. 4i, j). Upon HIMEC activation by TGF-β1, aldosterone also increased *Acta2* mRNA and protein levels, *Ccn2* and *Col1a1* mRNA levels, and MMP-2 activity in HIMEC (Fig. 4g–j). MRA by spironolactone repressed aldosterone-induced *Acta2* mRNA and protein levels, *Ccn2* mRNA and MMP-2 activity in HIMEC cells (Fig. 4g–j). These results show that aldosterone can induce fibrosis markers in CCD-18Co and HIMEC, two cell lines known to be myofibroblast progenitors, that was abolished by MR antagonist.

### Conditional MR genetic deletion in IEC did not prevent intestinal fibrosis

As MR is expressed in intestinal epithelial cells (IEC), which are involved in the pathogenesis of intestinal fibrosis via epithelial-mesenchymal transition (EMT), we next determined whether conditioned MR genetic deletion in IEC also prevents intestinal fibrosis (Supplementary Fig. 9a). Survival rate and colon weight/length ratio were higher in DSS treated mice with or without MR genetic deletion in IEC compared to DSS treated WT mice (Supplementary Fig. 9b, c). No significant difference was observed in body weight in DSS treated mice with or without genetic deletion compared to WT mice (Supplementary Fig. 9d). We next assessed ECM-associated genes and found that DSS administration increased *Mmp3* mRNA level while other ECM-associated genes were not differentially regulated between groups (Supplementary Fig. 9e). Genetic deletion of MR in IEC did not improve intestinal fibrosis in mice with chronic DSS.

### NGAL is up-regulated in the colon of mice with chronic DSS-induced intestinal fibrosis while MR genetic deletion and pharmacological antagonism prevents NGAL upregulation

**Fig. 5:**
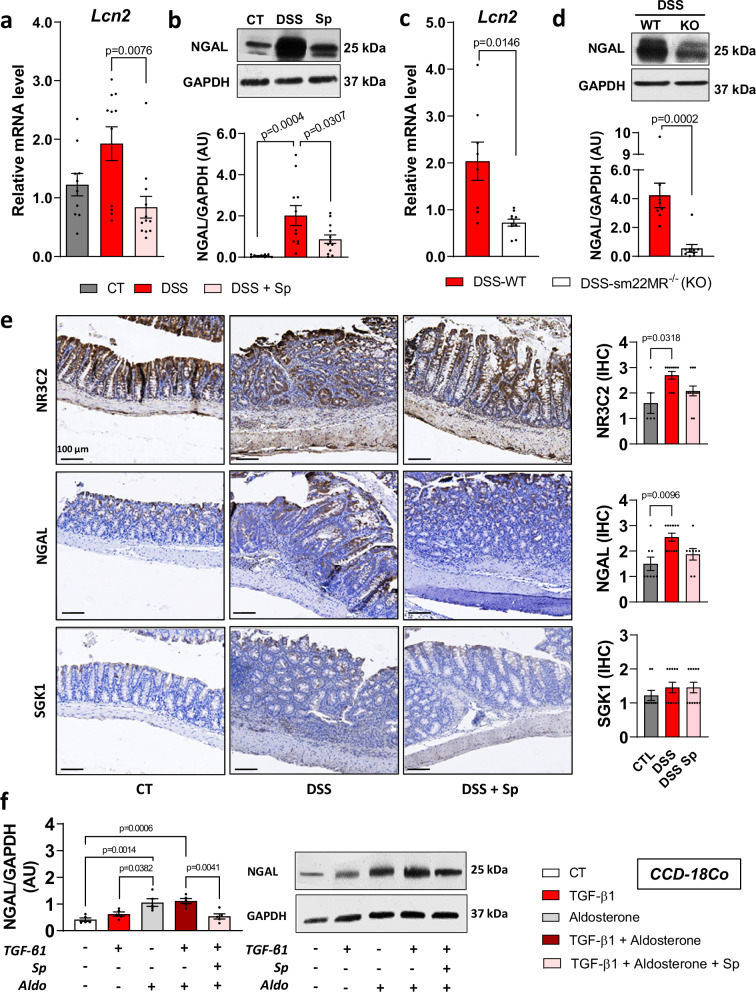
Neutrophil gelatinase-associated lipocalin (NGAL) level is increased in colon of mice with chronic colitis and in aldosterone-induced colon fibroblasts. **a, b** Male C57BL/6 J mice underwent 3 cycles of 2% dextran sulfate sodium (DSS) in their drinking water for 7 days, followed by 14 days of regular water (DSS, *n* = 11) for both first cycles and 7 days for the third cycle. whereas control mice received normal water (CT, *n* = 10). Mice were subjected to either a standard diet or diet supplemented with spironolactone (30 mg.kg^-1^; DSS+Sp, *n* = 12) throughout the experiment. **a** Relative mRNA and **b** protein expression of NGAL from colon of CT, DSS and DSS+Sp mice (*n* = 10 for CT, *n* = 11 for DSS, *n* = 12 for DSS+Sp; Kruskal-Wallis test with Dunn’s multiple comparisons test for mRNA expression; ordinary one way ANOVA with Tukey’s multiple comparisons test for protein expression). **c–d** Female C57BL/6 J mice underwent 3 cycles of 2% DSS in their drinking water for 7 days, followed by 14 days of regular water (DSS-WT, *n* = 8; DSS-sm22MR^−/−^, *n* = 10) for both first cycles and 7 days for the third cycle. **c** Relative mRNA and **d** protein expression of NGAL from colon of CT-WT, DSS-WT, DSS-sm22MR^−/−^mice (*n* = 8 for DSS = WT, *n* = 10 for DSS-sm22MR^−/−^; Two-sided unpaired t-test with Welch’s correction for mRNA epression; Two-sided Mann-Whitney test for protein expression). **e** Colon immunostaining of NR3C2, NGAL and SGK1 (*n* = 5 for CT, *n* = 10 for DSS, *n* = 12 for DSS+Sp; Kruskal-Wallis test with Dunn’s multiple comparisons test). **f** CCD-18Co cells were incubated with aldosterone (100 nM), spironolactone (10 µM) in response to TGF-β (10 ng/mL) for 24 h (*n* = 5 from independent experiments). Relative protein expression of NGAL from CCD-18Co cells lysates (*n* = 5 per group; ordinary one way ANOVA with Tukey’s multiple comparisons test). Data are presented as mean values +/− SEM.

Because we have previously shown that NGAL is an MR target in extra-intestinal fibrosis, we next quantified both mRNA (*Lcn2*) and protein NGAL levels in the colon of DSS-treated mice. We observed that both mRNA (*Lcn2*) and protein NGAL levels were higher in the colon of DSS-treated mice compared to control mice (Fig. 5a, b, e). These increases were prevented by spironolactone (Fig. 5a, b, e). We next determined NGAL levels in mice with sm22-MR deletion. SMC MR deletion decreased NGAL mRNA and protein levels in chronic DSS mice (Fig. 5c, d). We confirmed this finding by performing immunostaining in spironolactone-treated mice. DSS challenge upregulated MR and NGAL staining, while spironolactone-treated mice did not exhibit this increased (Fig. 5e). No difference in SGK1 immunostaining was observed among groups (Fig. 5e).

We then investigated in vitro the effects of MR agonist/antagonist on NGAL protein expression in CCD-18Co fibroblasts. The MR ligand aldosterone, alone or in combination with TGF-β1, upregulates NGAL protein expression in CCD-18Co cells, while TGF-β1 alone does not (Fig. 5f). The MRA by spironolactone decreased TGF-β1 and aldosterone-induced NGAL protein level in CCD-18Co cells (Fig. 5f).

### Spironolactone inhibits mineralocorticoid receptor binding on the Neutrophil gelatinase-associated lipocalin in TGF-β1-activated smooth muscle cells

**Fig. 6:**
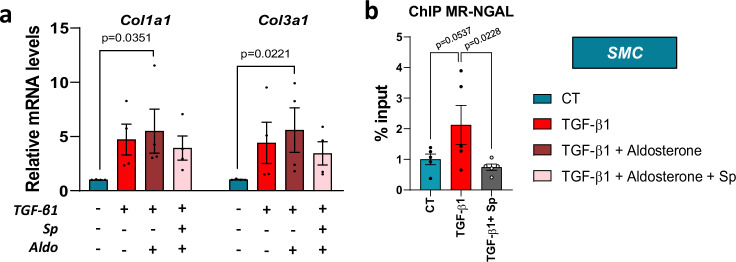
Spironolactone inhibits mineralocorticoid receptor (MR) binding on the neutrophil gelatinase-associated lipocalin (NGAL) in TGF-β1-activated smooth muscle cells (SMC). **a, b** Murine SMC were incubated with aldosterone (100 nM), spironolactone (10 µM) in response to TGF-β (10 ng/mL) for 24 h (*n* = 4 from independent experiments). **a** Relative mRNA expression of *Cola1* and *Col3a1* from SMC cells lysates (*n* = 4 per group; Kruskal-Wallis test with Dunn’s multiple comparisons test). **b** Chromatin immunoprecipitation (ChIP) followed by qPCR analysis (ChIP-qPCR) performed with an anti-MR antibody in SMC upon of TGF-β1 and spironolactone stimulation (*n* = 5 per group; Friedman test with Dunn’s multiple comparisons test). Data are presented as mean values +/− SEM.

We next determined by chromatin immunoprecipitation whether MR can bind to NGAL promoter in SMC. First, we observed that combination of aldosterone with TGF-β1 significantly upregulated *Col1a1* and *Col3a1* mRNA levels in SMC (Fig. 6a). We found that the binding of MR to the NGAL promoter was increased in the SMC upon TGF-β1 stimulation, without reaching statistical difference (p = 0.05), and was significantly reduced under MRA spironolactone treatment (Fig. 6b). These data suggest that MR participates to the transcriptional regulation of the NGAL promoter by MR in murine SMC.

### NGAL genetic deletion modulates colon fibrosis and TGF-β1 pathway in mice with chronic colitis

**Fig. 7:**
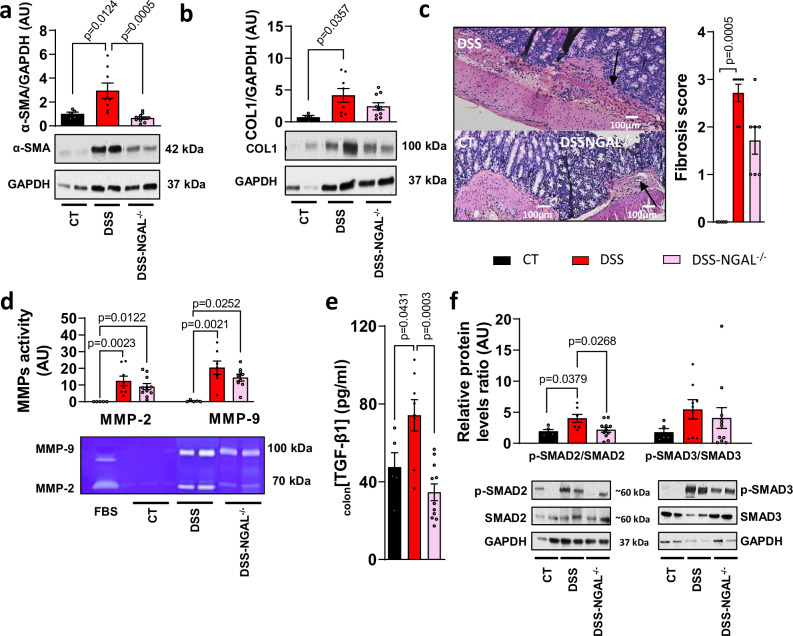
Neutrophil gelatinase-associated lipocalin (NGAL) genetic deletion decreased intestinal fibrosis and TGF-β1 pathways in mice with chronic colitis. Female C57BL/6 NGAL^−/−^ (*n* = 13) and DSS (*n* = 8) underwent 3 cycles of 2% DSS in their drinking water for 7 days, followed by 14 days of regular for both first cycles and 7 days for the third cycle. Control mice received normal drinking water (CT; *n* = 10). **a, b** Representative western blot and relative protein level of α-SMA (**a**, *n* = 5 for CT, *n* = 8 for DSS, *n* = 11 for DSS-NGAL^−/−^; ordinary one way ANOVA with Tukey’s multiple comparisons test) and COL1 (**b**, *n* = 4 for CT, *n* = 8 for DSS, *n* = 10 for DSS-NGAL^−/−^; Kruskal-Wallis test with Dunn’s multiple comparisons test) colon. **c** Hematoxylin-eosin-safran stained tissues in CT, DSS, DSS-NGAL^−/−^ groups and fibrosis score (*n* = 5 for CT, *n* = 7 for DSS, *n* = 7 for DSS-NGAL^−/−^; Kruskal-Wallis test with Dunn’s multiple comparisons test). **d** Representative gelatine zymography and quantification of colon MMP-9 (*n* = 5 for CT, *n* = 7 for DSS, *n* = 9 for DSS-NGAL^−/−^; Kruskal-Wallis test with Dunn’s multiple comparisons test) and MMP-2 (*n* = 5 for CT, *n* = 8 for DSS, *n* = 11 for DSS-NGAL^−/−^; Kruskal-Wallis test with Dunn’s multiple comparisons test) activity. **e** Colonic TGF-β1 level (*n* = 5 for CT, *n* = 8 for DSS, *n* = 11 for DSS-NGAL^−/−^; ordinary one-way ANOVA with Tukey’s multiple comparisons test). **f** Representative western blot and relative protein expression of phospho-SMAD 2/3 (p-SMAD2/SMAD2: *n* = 5 for CT, *n* = 7 for DSS, *n* = 11 for DSS-NGAL^−/−^, ordinary one way ANOVA with Tukey’s multiple comparisons test; p-SMAD3/SMAD3: *n* = 5 for CT, *n* = 8 for DSS, *n* = 11 for DSS-NGAL^−/−^; Kruskal-Wallis test with Dunn’s multiple comparisons test). Data are presented as mean values +/− SEM.

To elucidate the role of NGAL in vivo, we examined the effect of genetic deletion of NGAL in mice with chronic DSS (Supplementary Fig. 10a). NGAL genetic deletion increased the survival rate (Supplementary Fig. 10b), reduced colon weight/length ratio (Supplementary Fig. 10c) and colon MPO activity (Supplementary Fig. 10d) but had no effect on fecal calprotectin level (Supplementary Fig. 10e). We then observed that DSS upregulates *Il-6*, *TNF-α*, *MCP-1*, *Cxcl1*, *Cxcl8* and *Cxcr2* mRNA levels, while NGAL genetic deletion inhibited colon MCP-1 mRNA levels in mice treated with chronic DSS administrations compared to DSS mice (Supplementary Fig. 10f). Similarly, colon IL-6 production was up-regulated by DSS alone but not in DSS mice with NGAL genetic deletion (Supplementary Fig. 10g). As NF-κB regulates gene expression of many cytokines, we studied its activation. DSS treatment up-regulated p-NF-κB/NF-κB ratio while NGAL^−/−^ treated-DSS mice did not (Supplementary Fig. 10h). NGAL genetic deletion reduced chronic DSS-induced colon α-SMA and COL1 levels (Fig. 7a, b). Histologic analysis of tissue sections from mice deleted for NGAL exhibited a lower collagen deposition compared to the DSS WT group (Fig. 7c). NGAL genetic deletion did not impact MMP-2 and −9 activities (Fig. 7d). NGAL genetic deletion reduced colon TGF-β1 production (Fig. 7e) and SMAD2, but not SMAD3, signaling (Fig. 7f). NGAL genetic deletion reduced chronic DSS-induced colon *Nr3c2* and *Sgk1* mRNA levels (Supplementary Fig. 11)

### NGAL mediates the pro-fibrotic effect of MR activation and induces a pro-fibrotic phenotype in human colon fibroblasts and in mice with chronic DSS

**Fig. 8:**
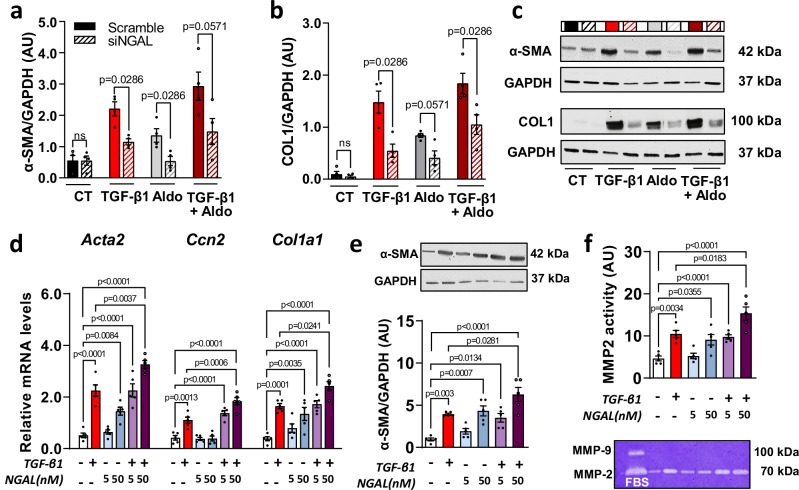
Neutrophil gelatinase-associated lipocalin (NGAL) mediates pro-fibrotic effect of mineralocorticoid receptor (MR) activation. **a, b** CCD-18Co cells silenced for NGAL with siNGAL were incubated with aldosterone (Aldo, 100 nM) with or without TGF-β (10 ng/mL) for 24 h (*n* = 4 from independent experiments). Relative protein levels of α-SMA (**a**), COL1 (**b**) and representative image of western blot (**c**) from CCD-18Co cells lysates (*n* = 4 per group; Two-sided Mann-Whitney test). **d–f** CCD-18Co cells were incubated with increasing concentration of recombinant hNGAL (5 ng/ml and 50 ng/ml; *n* = 5 from independent experiments). **d** Relative mRNA level of *Acta2*, *Ccn2* and *Col1a1* from CCD-18Co cells lysates (*n* = 5 per group; ordinary one way ANOVA with Tukey’s multiple comparisons test). **e** Representative and relative protein expression of α-SMA from CCD-18Co cells lysates (*n* = 5 per group; ordinary one way ANOVA with Tukey’s multiple comparisons test). **f** Representative gelatine zymography and quantification of MMP-2 activity from CCD-18Co cell lysates (*n* = 5 per group; ordinary one way ANOVA with Tukey’s multiple comparisons test). Data are presented as mean values +/− SEM.

To investigate how NGAL mediates the MR-induced pro-fibrotic effects in fibroblasts, NGAL silencing was performed in CCD-18Co cells (Fig. 8a–c, Supplementary Fig. 12). First, we observed that NGAL silencing reduces TGF-β1- and/or aldosterone-induced NGAL protein level in CCD-18Co (Supplementary Fig. 12a). Silencing of NGAL blunts TGF-β1- and/or aldosterone- induced *Acta2* and *Col1a1* mRNA levels (Supplementary Fig. 12b, c), and α-SMA and COL1 protein levels (Fig. 8a-c) in CCD-18Co.

To study whether NGAL directly exerts pro-fibrotic effect in CCD-18Co, we incubated CCD-18Co with increasing concentrations of NGAL. NGAL treatment upregulates TGF-β1 mRNA levels in CCD-18Co cells (Supplementary Fig. S13). NGAL has an additional effect to TGF-β1 to induce ECM-associated mRNA levels such as *Acta2*, *Ccn2* and *Col1a1* (Fig. 8d), increase α-SMA protein level (Fig. 8e) and MMP-2 activity (Fig. 8f).

**Fig. 9:**
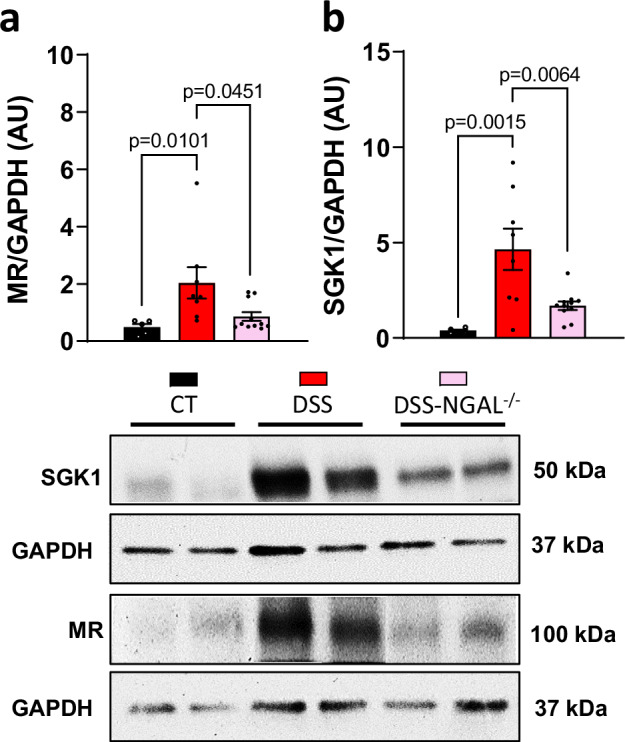
Effect of genetic deletion of neutrophil gelatinase-associated lipocalin (NGAL) on colon mineralocorticoid receptor (MR) and SGK1 expression in female mice with dextran sulfate sodium (DSS)-induced colitis. Female C57BL/6 DSS-NGAL^−/−^ (*n* = 13) and DSS (*n* = 8) underwent 3 cycles of 2% DSS in their drinking water for 7 days, followed by 14 days of regular water for both first cycles and 7 days for the third cycle. Control mice received normal water (CT; *n* = 10). **a, b** Representative western and relative protein level of MR (**a**) and SGK1 (**b**) colon (*n* = 5 for CT, *n* = 8 for DSS, *n* = 11 for DSS-NGAL^−/−^; Kruskal-Wallis test with Dunn’s multiple comparisons test). Data are presented as mean values +/− SEM.

We next determined in vivo whether NGAL genetic deletion in mice with chronic DSS affectes colon MR expression and SGK1, a MR target. We observed that genetic deletion of NGAL inhibits DSS-induced colon MR (Fig. 9a) and SGK1 expression (Fig. 9b).

## Discussion

**Fig. 10:**
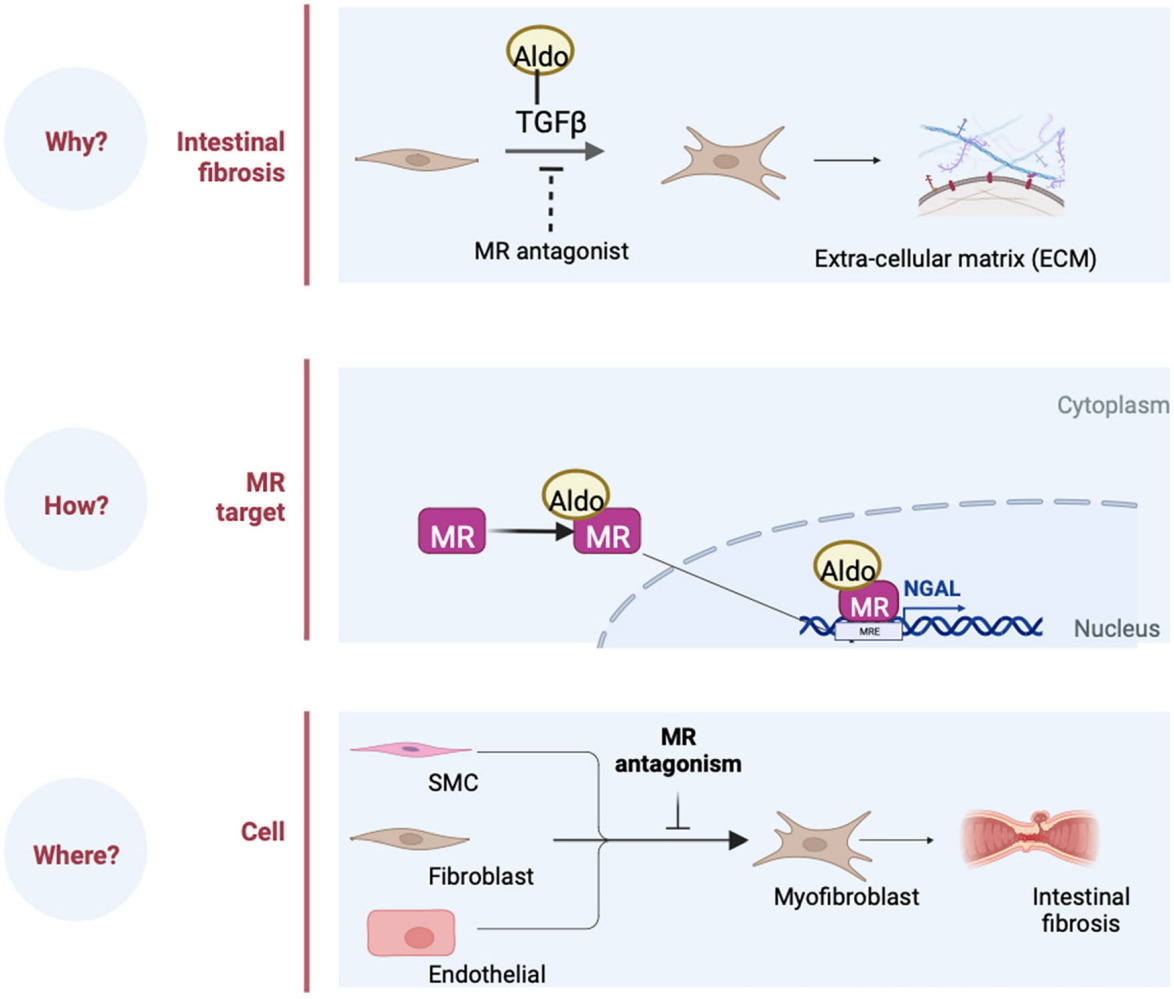
Mineralocorticoid receptor (MR) activation promotes experimental intestinal fibrosis through neutrophil gelatinase-associated lipocalin (NGAL) pathway. Aldosterone (Aldo), MR ligand promotes fibroblast activation while MR antagonism by spironolactone inhibited it. NGAL is a downstream MR target in intestinal fibrosis. MR antagonism reduced extracellular matrix production in smooth muscle cells (SMC), intestinal fibroblasts, and intestinal endothelial cells. Created in BioRender^67^.

We describe here a pre-clinical study demonstrating that pharmacological MRA and genetic SM specific MR deletion inhibits intestinal fibrosis in an established model of IBD. In addition, the present study highlights the involvement of NGAL to mediate MR pro-fibrotic effects in this context. These results are summarized in the Fig. 10.

Intestinal fibrosis is a common complication of IBD that can cause bowel strictures and stenosis and may require surgical procedures. There is no specific treatment for intestinal fibrosis, and anti-inflammatory therapy does not limit the progression of intestinal fibrosis. For this reason, a therapy for intestinal fibrosis is an unmet need and a challenge in IBD^15^.

Intestinal fibrosis is characterized by an excessive accumulation of ECM, and many cellular types are implicated in this process^18^. Among these cells, myofibroblasts are the final effector cells. While these cells mainly derived from resident fibroblasts and SMC, they can also originate from epithelial or endothelial cells through epithelio- or endothelio-mesenchymal transition. The various cell types interact with each other and contribute to the development of intestinal fibrosis^19^.

Fibrosis is a complication in many organs and intestinal fibrosis shares similarities in pathogenic pathways with extra-intestinal organs. As the MR is expressed in various cell types and organs^9^ and is a therapeutic target for cardiovascular and renal inflammation and fibrosis^20,21^, it may represent a good therapeutic target for intestinal fibrosis. MR and its ligand, aldosterone, are the main modulators of hormone-regulated renal sodium absorption. In addition, MRA decreases inflammation and fibrosis in a variety of organs and in various pathophysiological settings^22,23^. Of note, MR is highly expressed in the colon^9^ (both in epithelial and SMC^10^).

We demonstrated that TGF-β1, a core cytokine in intestinal fibrosis, induces MR mRNA expression in fibroblastic CCD-18Co cells and is associated with up-regulation of ECM-associated markers. In vivo, we also observed a MR overactivation in the colon of mice with chronic DSS-induced intestinal fibrosis: mice exhibited higher plasma aldosterone level associated with a higher colon expression of MR and its target, SGK1. An increase in SGK1 level has also been described in the intestinal epithelium from CD patients^24^, which may result from MR activation.

To further explore the effects of MRA in intestinal fibrosis, we assessed the effect of an MR antagonist, spironolactone. We showed that spironolactone reduced aldosterone-induced fibrosis-related markers in human fibroblastic CCD18-Co cells. As intestinal myofibroblasts are derived from a variety of cell types, we used a second cell type to confirm our findings^17,25^. We did not assess endoMT in the murine models because in vivo assessment of EndoMT required endothelial lineage tracing mice. As described in the study from Rieder et al.^19^, endothelial cells lose endothelial markers during EndoMT which thus prevents their monitoring. Rieder et al. developed an elegant technique where they used transgenic mice in which GFP is expressed under the control of an endothelial cell–specific promoter. By this technical approach, cells derived from endothelial cells can be detected by GFP expression and can still be recognized, even after transformation cells, by the co-expression of mesenchymal markers and GFP. As we do not have access to this EndoMT reporter mice, we used in vitro assessment of EndoMT in HIMEC in response to TFG-β instead of this technique requiring double transgenic mice. We observed that aldosterone induces fibrosis-related markers in primary culture of HIMEC, while spironolactone inhibits them. Importantly, pharmacological MRA by spironolactone blunts fibrosis development as shown by decreased ECM-associated markers in mice with chronic DSS. These results echo findings made in the context of cardiac and renal fibrosis^26^.

In mice with chronic DSS, NF-κB activity is upregulated compared to controls but not in mice receiving spironolactone treatment. This is in accordance with a study performed in human mononuclear cells showing that spironolactone inhibits NF-κB activity through reduced phosphorylation of IκB^27^. Similarly, spironolactone inhibits IκB kinase (IKK) in RAW 264.7 macrophage-like cells and mouse peritoneal macrophages in response to LPS^28^. It is in a particular interest in IBD. In chronic colitis models, inflammation and fibrosis persist as demonstrated by us and others^29^. In IBD patients, control of inflammation by anti-inflammatory therapies such as anti-TNF is not sufficient to limit the development of intestinal fibrosis. In contrast, MRA can target fibrosis and inflammation. As mentioned above, spironolactone target NF-κB activity, which is itself involved in the regulation of pro-inflammatory markers such as pro-inflammatory cytokines.

We performed chronic colitis in a second model of intestinal fibrosis in mice using TNBS administration. In this experimental series, we cannot conclude to the effect of spironolactone because our mice were resistant to TNBS-induced inflammation and fibrosis. Resistance of C57BL6 mice to TNBS challenge is described in some studies^30^ while other teams succeeded to induce inflammation and fibrosis to this mice strain^30^. We chose to avoid the TNBS model with a pre-sensibilization for ethics reasons because of its high mortality rate. We thus switched to the Sprague-Dawley rats and induced a chronic colitis model in rats by TNBS administration. TNBS administration significantly reduced body weight and upregulated mRNA levels coding for fibrosis markers such as *Acta2*, *Col1a1* and *Vim*. Spironolactone treatment had no significative effect on the studied parameters compared to rats with chronic TNBS colitis.

We speculated that the absence of spironolactone effect in TNBS may result from the used dose or the treatment duration. Indeed, Sehirli et al. used spironolactone at 80 mg.kg^-1^ for 7 days in Sprague-Dawley rats with acute TNBS-induced colitis and they observed that spironolactone reduced inflammation with a lower NF-κB activity and colon MPO^31^. We used a lower dose (30 mg.kg^-1^) to match the dose used in mice. A second study investigated the effect of spironolactone in rats with chronic TNBS-induced colitis and they observed 44% mortality in rats at a 20 mg.kg^-1^ and a decreased mortality with lower doses of spironolactone but the lower doses did not reduce the development of fibrosis^32^. We did not observe higher mortality in our TNBS series between TNBS rats with or without spironolactone and we used a higher number of rats per experimental group (12 instead of only three in Johnson’s study^32^). In our experiments with chronic TNBS colitis in mice, the spironolactone treatment lasts 9 weeks while only 31 days in mice and it may explain the discrepancy between both colitis models.

Heterogeneity is also described in IBD patients and in experimental models of IBD and it challenges the identification of biomarkers in response to therapies^33^. Villablanca’s team proposed a very elegant way to stratify UC patients^34^. They compared human data sets with a longitudinal transcriptome profile of murine DSS-induced colitis^34^ and they succeeded in separating UC patients in two distinct clusters with this molecular signature (defined as UC1 and UC2). Of note, UC1 patients presented a lower response to therapies compared to UC2 patients (10% *vs*. 70% to anti-TNF therapies) and UC2 are characterized by a high expression of NOX1, NR3C2 and PARM1 compared to UC1.

Among the chemically induced colitis models, the two main and most widely used in drug testing are the TNBS and DSS experimental models. Differences of responses to drugs is already described in IBD models. For example, dexamethasone treatment is more effective in male mice with DSS compared to male mice with TNBS^35^. Similarly, IEC expression of TNFAIP3 protects mice from DSS- but not TNBS-induced colitis^36^. This difference may also be explained by the cytokine profiles induced by both chemically-induced colitis models. While chronic DSS is more characterized by Th2-mediated inflammatory response with higher IL-4 and IL-10, chronic TNBS is more characterized by a Th1-Th17 profile^37^. Bilsborough et al. compared by literature search the respective value of murine colitis models, including chronic DSS and TNBS models to predict clinical efficacy of therapeutics in UC patients and they conclude that none of these models were consistently superior in predicting efficacy^38^.

Intestinal fibrosis is defined by an excessive ECM from activated myofibroblasts. Since MR is expressed in SMC^10^ and SM expansion is a major feature in intestinal fibrosis^17^, we also investigated the cell-specific role of MR in intestinal fibrosis. Specific genetic deletion of MR in SMC reduced chronic DSS-induced intestinal fibrosis in mice with a lower TGF-β1 signaling and collagen deposition. These data further support a role of MR in intestinal fibrogenesis. The effect of MR genetic deletion in SMC appears more pronounced in female mice than in male mice, which still need to be confirmed on larger datasets. A sexual dysmorphism has already been observed for MR-mediated injuries. For example, the MR antagonist, eplerenone, is only beneficial in infarct male mice while myeloid MR genetic deletion is protective in both male and female mice^39^. MR drives vascular dysfunction induced by cardiovascular risk factors via sexually dimorphic mechanisms related to MR/estrogen receptor α interactions and the MR protein level itself is sex-dependent^40^. MR genetic deletion in SMC also improved mice survival to chronic DSS and may result from a general better well-being and a higher body weight. In the literature, body weight loss is even used to evaluate the severity of colitis in mice^41^. Undernutrition may also result from inflammation-induced intestinal malabsorption and fibrosis-induced stenosis and is associated with a higher mortality rate in mice and also in patients with IBD^42^.

Understanding the molecular mechanisms associated to MR pathway activation is crucial to identify potential novel therapeutic targets downstream of MR. As previously demonstrated, NGAL plays a critical role as a novel MR-modulated target in the pro-inflammatory and pro-fibrotic processes induced by mineralocorticoids^43^, we therefore investigated its potential involvement in the context of intestinal fibrosis. NGAL is a 25-kDa glycoprotein of the lipocalin superfamily expressed by a variety of cell types, including epithelial cells, endothelial cells, SMC, and various immune-cell populations, such as neutrophils, macrophages, and dendritic cells^43^. NGAL is considered as a disease marker for active IBD^12^ and described as the top-ranked upregulated gene in a recent meta-analysis of microarrays from inflamed tissues biopsies from UC patients^44^. In addition, fecal NGAL is commonly used as a pro-inflammatory marker in colitis^13^.

Our study showed that recombinant human NGAL increased ECM-associated markers such as TGF-β1 and COL1 in human CCD-18Co cells, as we previously observed in human cardiac fibroblasts^14^. In the present study, we also show that siRNA targeting NGAL down-regulates ECM markers such α-SMA or COL1, in aldosterone-induced human fibroblast cell line in response or not to TGF-β1. These results provide ex vivo data concerning the role of MR through NGAL, and it demonstrates in vitro that lack of NGAL prevents aldosterone-mediated effects on ECM production. In addition, aldosterone up-regulated NGAL expression through MR activation in CCD-18Co cells. As previously demonstrated in cardiac fibroblasts^45^, we showed that NGAL knockdown abolished aldosterone-induced collagen I expression in CCD-18Co cells. We have previously reported a mineralocorticoid-responsive element in the NGAL promoter that is involved in aldosterone-MR-mediated mouse NGAL promoter activity using a luciferase assay^46^. Here, we showed by ChIP that MR antagonist inhibits MR binding on the NGAL promoter in TGF-β1-activated SMC.

In vivo, we observed higher fecal level and colon expression of NGAL in mice with chronic DSS-induced intestinal fibrosis. Pharmacological MRA and genetic MR deletion inhibited colon NGAL overexpression in mice with chronic DSS-induced intestinal fibrosis. To test the potential role of the MR target NGAL in the disease process of intestinal fibrosis, chronic colitis was also induced in NGAL deleted mice. Loss of NGAL abolished the colon MR activation in mice with DSS-induced chronic colitis. TGF-β1/SMAD pathway is a key mediator in a variety of organ fibrosis and is described as the most potent inducer of fibroblasts activation^47^. Our study showed that NGAL deletion abolished the colon TGF-β1/SMAD pathway activation in mice with chronic DSS-induced intestinal fibrosis. Importantly NGAL deletion was associated with a marked decrease in α-SMA expression and collagen deposition in the colon of chronic DSS treated mice. Taken together, these observations highlight a common pathway involved in the regulation of MR and NGAL and reinforce our hypothesis that MR activation promotes intestinal fibrosis through the NGAL pathway. Of note, NGAL deletion in mice was associated with a decreased colon MR and SGK1 expression. As NGAL can activate the transcription factor NF-κB^45^, which is able to bind and regulate MR^48^, this may contribute to amplify a vicious profibrotic circle involving both MR and NGAL. As previously demonstrated in cardiac fibroblasts^45^, we showed that NGAL knockdown abolished aldosterone-induced collagen I expression in CCD-18Co cells. NGAL modulates NF-κB^45^, which is known to regulate the gene expression of *Col1a1*. The blunting of *Col1a1* expression upon aldosterone stimulation with or without TGF-β by NGAL knockdown may thus occur through NF-κB inhibition. Another putative mechanism would be the involvement of NGAL in modulating the IL-4-STAT6 pathway, as we previously demonstrated in a model of kidney fibrosis^49^.

The role of NGAL in colitis is controversial. Singh et al. have reported a more pronounced colitic response to acute DSS treatment in NGAL-deleted mice^50^ while we observed that NGAL deletion reduced chronic DSS-induced colitis development. As reported by Singh et al^50^., we also observed a reduced colon MPO activity in DSS-treated NGAL^−/−^ mice. While both studies used the same protocol (C57BL6 mouse strain, same supplier of DSS, similar dose of DSS 1.8 *vs*. 2 %), they used acute colitis model with one DSS cycle while we used a chronic DSS model with three cycles and it may explain this discrepancy. It may be also dependent of the cell type in colitis. Indeed, a study has showed that NGAL prevents LPS-induced intestinal inflammation by enhancing macrophage-mediated bacterial clearance^51^. It has been reported that the main source of NGAL in the gut are IEC and myeloid cells. NGAL is induced during intestinal inflammation, which leads to high levels of NGAL in intestinal mucosa and feces^52^. In active IBD patients, serum and fecal NGAL are massively increased^12^. To assess whether the deficiency of NGAL in SMCs attenuates the deleterious effects of MR in SMCs, we first used a global NGAL^−/−^ mice. In the experiment using NGAL^−/−^ mice, we found that NGAL is a key component in intestinal inflammation and fibrosis processes. NGAL genetic deletion reduced signaling pathways such as pro-inflammatory cascades as shown by a reduced colon weight/length ratio, a lower colon MPO activity and lower mRNA levels of cytokines such as Il-6, TNF-α, MCP-1 including MR-mediated inflammatory pathways. The use of siRNA targeting NGAL reduced aldosterone-induced α-SMA mRNA levels in the human intestinal fibroblast cell line CCD-18Co. NGAL genetic deletion also reduced pro-fibrotic pathways with a reduced colon α-SMA expression and a lower SMAD signaling activation.

Similarly, our in vitro data demonstrated a crucial role of NGAL in CCD-18Co. Indeed, siRNA against NGAL reduced aldosterone-induced α-SMA mRNA levels in the human intestinal fibroblast cell line CCD-18Co. Of note, siRNA against NGAL also reduced TGF-β induced α-SMA mRNA in CCD-18Co cells showing that NGAL can also act independently of MR signaling.

Further investigations are now required to better understand the role of the MR and its target NGAL in patients with IBD. As the intestine is the largest interface between the host and the environment, it may be of interest to explore crosstalk between the MR and the gastrointestinal exposome. This may be especially relevant in the renewed interest in dietary research in IBD. Indeed, dietary modulation of the MR, in particular in the case of obesity-induced MR activation, has been reported in extra-intestinal fibrosis^53^ and may also occur in IBD patients.

Here, we show that TGF-β alone or in combination with aldosterone induced NGAL expression in fibroblasts in accordance with what we have previously shown in human cardiac fibroblasts upon aldosterone treatment^54^. We also reported that NGAL is a direct target of MR as MR binds to the NGAL promoter and activates promoter activity upon aldosterone stimulation^54^. In conclusion, we demonstrate that MR is involved in intestinal fibrosis induced by chronic DSS administration in vivo and we report that NGAL plays a key role in mediating MR-induced pro-fibrotic effects. Intestinal fibrosis is an unmet medical need, and the repositioning of MR antagonists already available in other medical applications may also open new therapeutic avenues in IBD.

## Methods

### Mouse experiments

#### Ethics

All animal care protocols and experiments were conducted in accordance with the European directive for the use and care of laboratory animals (2010/63/UE) and received the approval of the local animal ethics committee (Comité National de Réflexion Ethique sur l’Expérimentation Animale) and of the ministerial committee for animal experimentation (#16184-2018071711054339). All mice and rats were reared under conventional pet facility (at 22 ± 1 °C, 12 h light-dark cycle, dark phase: 9.30pm-9.30am) with ad libitum access to a regular chow diet (SAFE, Augy, France) and were co-housed 5 per cage. The study was carried out in compliance with the ARRIVE guidelines. At the end of the animal experiment, mice were killed by an overdose of anaesthesia (ketamine/ Xylazine solution, 40 and 1 mg. kg−1, respectively).

#### Transgenic models

We used male and female mice between 8 and 9 weeks of age. The sm22-MR^−/−^ female and male mice were generated by crossing floxed MR mice (MRfl/fl) with transgenic mice expressing Cre recombinase under the control of the regulatory elements of the mouse SM22alpha gene^55^. Mice were confirmed for their sm22-MR^−/−^ genotype by PCR (Supplementary Table 1, Supplementary Fig. 14). Sm22-MR^−/−^ have a normal phenotype with no difference for growth, feeding and levels of electrolytes compared to WT mice^14,56–59^. NGAL^−/−^ C57BL/6 J female mice were generated^60^, genotyped, and obtained from Cordeliers Research Center (INSERM U1138, Paris, France). The Villin-CreERT2 MRfl male mice were generated by crossing floxed MR mice (MRfl/fl) with transgenic mice expressing Cre recombinase under the control of the Villin gene promoter^61^ and were induced by a tamoxifen injection before DSS cycles.

#### Dextran sodium sulfate-induced chronic colitis

Chronic colitis was induced by 3 cycles of 1 to 2% dextran sodium sulfate (DSS, 35–50 kDa; MP Biomedicals, Illkirch, France) dissolved in drinking water as published protocol^62^. A cycle of DSS was defined as 1 week of DSS administration followed by 2 weeks of recovery period on normal drinking water except for the third cycle of 1 week of DSS administration followed by a recovery period of 1 week. Throughout the duration of the experiment, normal drinking water was given to control mice. After 8 weeks from DSS challenge, all mice were killed under Ketamine/Xylazine anaesthesia (experimental flow chart Fig. 1a).

#### Pharmacologic MR antagonism in DSS colitis

Chronic DSS colitis were induced in 8-week-old C57BL/6 J male mice purchased from Janvier (Le Genest St Isle, France). MRA was investigated by dietary administration of spironolactone (Fagron, Belgium) at 30 mg.kg^−1^ *ad libitum* in colitic male mice (Supplementary Fig. 1a). Control animals received standard diet (SAFE, Augy, France). Body weight was recorded throughout the experiment (Supplementary Fig. 1b).

### Cell experiments

#### Cell culture

We used CCD-18Co, a human cell line exhibiting fibroblast morphology that was isolated from the normal colon tissue of a 2.5-month-old, Black, female and this cell line was obtained from ATCC (Washington, USA). We also used Human Intestinal Microvascular Endothelial (HIMEC) cells obtained from ScienCell Research Laboratories (USA). CCD-18Co were cultured in Minimum Essential Medium Eagle (MEM; Eurobio, France) supplemented with 10% of fetal bovine serum (Gibco, USA), 1% of penicillin/streptomycin (Dutscher, France), 2 mM of L-glutamine (Dutscher, France) and 1% of MEM non-essential amino acid (Sigma, USA). Human primary Intestinal Microvascular Endothelial (HIMEC) cells were purchased from ScienCell Research Laboratories (Carlsbad, USA) and cultured in endothelial cell medium supplemented with 5% of fetal bovine serum, 1% of penicillin/streptomycin and 1% of endothelial cell growth factor (EGF; ScienCell Research Laboratories, USA)^63–65^. C57BL/6 Mouse Primary Colonic Smooth Muscle Cells (SMCs) were obtained from CellBiologics (Chicago, USA). SMCs cells were cultured in Smooth Muscle Cell Medium (CellBiologics, Chicago, USA) supplemented with fetal bovine serum, antibiotic-antimycotic solution, hydrocortisone, fibroblast growth factor (FGF), epidermal growth factor (EGF), insulin, and glutamine. SMCs were proliferated in flasks or petri dishes coated with gelatin-based coating solution (CellBiologics, Chicago, USA).

Cells were maintained in 5% CO_2_ air-humidified atmosphere at 37 °C. All experiments were performed using cells within 4 to 12 passages. Mycoplasma detection was routinely examined (supplemental method).

#### Cell transfection

CCD-18Co cells were seeded into 12-well plates. At 80% cell confluence, cells were transfected by a pool of four NGAL siRNA targeting NGAL (L-003679-00-0005) or by non-targeting siRNA (scramble condition, D-001810-10-05) purchased from Horizon Discovery (UK). All transfection assays were carried out using Lipofectamine 3000 reagent (Invitrogen, USA) as per the manufacturer’s instructions.

#### Cell treatment

Cells were incubated with 10 ng/mL of human recombinant TGF-β1 (PeproTech, USA) for 24 h to induce cell differentiation into activated fibroblasts. Cells were also induced with the MR ligand aldosterone (100 nM; Sigma-Aldrich, USA), the MR antagonist spironolactone (10 µM; R&D Systems, USA) or recombinant human NGAL (hNGAL; 5 to 50 ng/mL; R&D Systems, USA) for 24 hours. After treatment, supernatants were collected and stored at −80 °C until analysis. After three washes with PBS, cells were lysed in 200 µl of Cellytic™ buffer (Sigma-Aldrich, USA) for protein analysis or lysed in 500 µL of Trizol reagent (Invitrogen, USA) for RNA analysis.

#### Chromatin Immunoprecipitation PCR (ChIP)

SMCs were cultured as described above until confluence and fixed with 1% formaldehyde (Thermo Scientific, Wakthal, USA). 200 µg of sonicated chromatin extract were incubated with pre-conjugate MR antibody (Millipore) to Protein A + G magnetic beads (Merck) overnight. MR binding on NGAL promoter was determined by qPCR of the enriched DNA (after immunoprecipitation) and of input samples (without immunoprecipitation) using the following primers: F > 5′-GGTATTGGACACTTCCAGGATAATC-3’ and R > 5′- AAGCCAAAGCCATCTGAACACCAGA-3’.

#### PCR arrays

Primers design and PCR arrays assays were performed by the PRIMACEN platform. Primers were designed with the software Primer Express (v3.0.1; ThermoFischer Scientific) using nucleotide sequences from the NCBI Pubmed database. Primer pairs were ordered from Integrated DNA Technologies and validated by linear regression of serial dilution data. The list of primer pair sequences is available in Supplemental Table 2.

The determination of the expression level of genes was done by quantitative PCR in 384-well plates with a 5 µL reaction volume in the presence of 1 µL Fast SYBR Green PCR Mastermix (Thermofisher, cat. 4,385,612) containing pre-set concentrations of dNTPs, MgCl2 and the SYBR Green reporter dye along with a set of specific primers. The distribution of cDNA samples and reaction mixes was performed by a Bravo Automated Liquid Handling system (Agilent). The PCR reaction was conducted with a QuantStudio 12 K Flex thermal cycler (ThermoFisher scientific). Two technical replicates per animal and per gene of interest (GOI) were averaged prior to ΔCq calculation. The average Cq value of three housekeeping genes (HKG) was used to calculate ΔCq. Efficiency for all primer pairs was above 95% in order to apply confidently the 2(−ΔΔCq) method. This method provides a relative quantification of the expression of a gene within an experimental condition.

#### Gene expression by quantitative RT-qPCR analysis

The expression level of genes specific for inflammation and fibrosis were evaluated as described in supplemental method.

#### Analysis of protein level by Western Blot

The level of protein involved in inflammation, fibrosis, and MR activation were evaluated as described in Supplemental method.

#### Matrix metalloprotease (MMP) activity by gelatin zymography

MMP-2 and −9 activity was assessed by gelatin zymography. 10 µg of protein sample was diluted in a loading buffer containing: 1 M tris (pH 6.8), 2% Sodium Dodecyl Sulfate (SDS; Sigma-Aldrich, USA), 20% glycerol (Sigma-Aldrich, USA) and 0.02% bromophenol blue (Biorad, France). Fetal bovine serum containing MMP-9 and MMP-2 activity was used as a positive control (Gibco, USA). Samples were separated by electrophoresis on 8% (w/w) acrylamide gels containing 1% of gelatin (Sigma-Aldrich, USA). After 3 washes of 10 min with a washing buffer (2.5% Triton X-100) at room temperature, gels were incubated overnight at 37 °C with a buffer solution containing: 50 mM Tris-HCl at pH=7.5, 5 mM CaCl_2_, and 0.1% Triton X-100. Gels were incubated with a blue Coomassie coloration solution (0.1% Blue Coomassie, 40% methanol and 10% acetic acid) for 30-60 min at room temperature. MMP-2 and MMP-9 activity appeared as negative staining and were quantified using ImageJ 1.51 software (NIH, USA).

#### Colon myeloperoxidase assay

Colon MPO was assessed as described in Supplemental method.

#### Colon cytokine production by ELISA

Colon samples were homogenized mechanically in 500 µL of PBS with 1% of protease and phosphatase inhibitors (Sigma Aldrich, USA) by a bead-beating step (5 min of beating after addition of 2 steel beads per sample) using the TissueLyser LT machine (Qiagen, UK). Homogenates were centrifuged (12000 g, 15 min, 4 °C) and supernatants were collected. IL-6, IL-17, and TGF-β1 were measured in duplicate by ELISA according to the manufacturer’s protocol (R&D Systems, Minneapolis).

#### Histology

Colon samples were fixed in 4% formaldehyde and embedded in paraffin wax blocks. Sections of 4 µm were cut with a microtome. Tissue fibrosis was evaluated with Sirius red coloration, which allows collagen fiber coloration. Quantification of fibrosis was performed with QuPath software using pixel classification of stained areas. Briefly, a set of images were used for the creation of the pixel classifier. A trained pathologist annotated the stained areas in order to train the algorithm to recognize red staining (collagen) based on an artificial neural network. Automatic detection of fibrosis was then applied to the following samples. Quantification is expressed as a percentage of stained area of total tissue detection. Disease activity score was evaluated on HES coloration by a trained pathologist according to Nancy grade that is routinely used for ulcerative colitis^66^.

#### Immunohistochemistry

Immunohistochemical analyses were carried out using standard procedures. Slides were deparaffinized and pretreated using heat-mediated antigen retrieval in Benchmark Ultra CC1 buffer (Roche). Slides were then incubated with primary antibody at the following dilutions during 30 min at 37 °C in a humidity chamber (1/100 for SGK1 (SC05-71-Invitrogen), 1/1000 for NGAL (ab216462-abcam) and 1/500 for NR3C2 (PA5-116990-Invitrogen)). After rinsing in washing buffer, slides were incubated with secondary antibody (Polink-1 HRP Rabbit DAB Detection Kit D13-18-Origene) for 30 min at 37 °C in a humidity chamber. DAB chromogen-based revelation was carried out following manufacturer's instruction. A trained pathologist evaluated staining intensity using a semi-quantitative scale (0: no staining; 1: low staining visible at high magnification; 2: moderate staining visible at low magnification; 3: strong staining recovering nucleus).

#### Plasma aldosterone assay

Plasma aldosterone level was assessed by ELISA according to the manufacturer’s protocol (R&D Systems, Minneapolis).

#### Fecal Calprotectin and NGAL assay

Feces samples were weighed and homogenized mechanically in 600 µL of PBS with 1% protease and phosphatase inhibitors (Sigma-Aldrich, USA) by a bead-beating step (5 min of beating after addition of 2 steel beads per sample) using the TissueLyser LT machine (Qiagen, UK). After centrifugation (12000 g, 15 min, 4 °C) supernatants were collected and calprotectin or NGAL were assessed in duplicate by ELISA according to the manufacturer’s protocol (R&D Systems, Minneapolis).

#### Statistical analysis

All data were analysed using GraphPad Prism 8.0 software (GraphPad Software Inc., San Diego, CA, USA). Data were expressed as mea*n* ± standard error to mean. Inter-individual comparisons between two groups were performed with a parametric Student *t* test or non-parametric Mann-Whitney test, and one-way ANOVA followed by Tukey or Bonferroni post-tests for more than two groups. A P value  <  0.05 was considered significant.

### Reporting summary

Further information on research design is available in the Nature Portfolio Reporting Summary linked to this article.

## Supplementary information

Supplementary Information

Reporting Summary

## Source data

Source Data

## Data Availability

All processed data used to make figure panels are available in a source data file. The source data file also contains the all the required data to replicate our statistical testing of hypotheses. Source data are provided with this paper.
