# Supplementary material for: Mineralocorticoid receptor activation contributes to intestinal fibrosis through neutrophil gelatinase-associated lipocalin in preclinical models
Source: Nat Commun. 2025 Jul 9;16:6318. doi: 10.1038/s41467-025-61401-0 (PMC12241341; doi:10.1038/s41467-025-61401-0)
Supplement: Supplementary file 1 — Supplementary Information [file 41467_2025_61401_MOESM1_ESM.pdf]

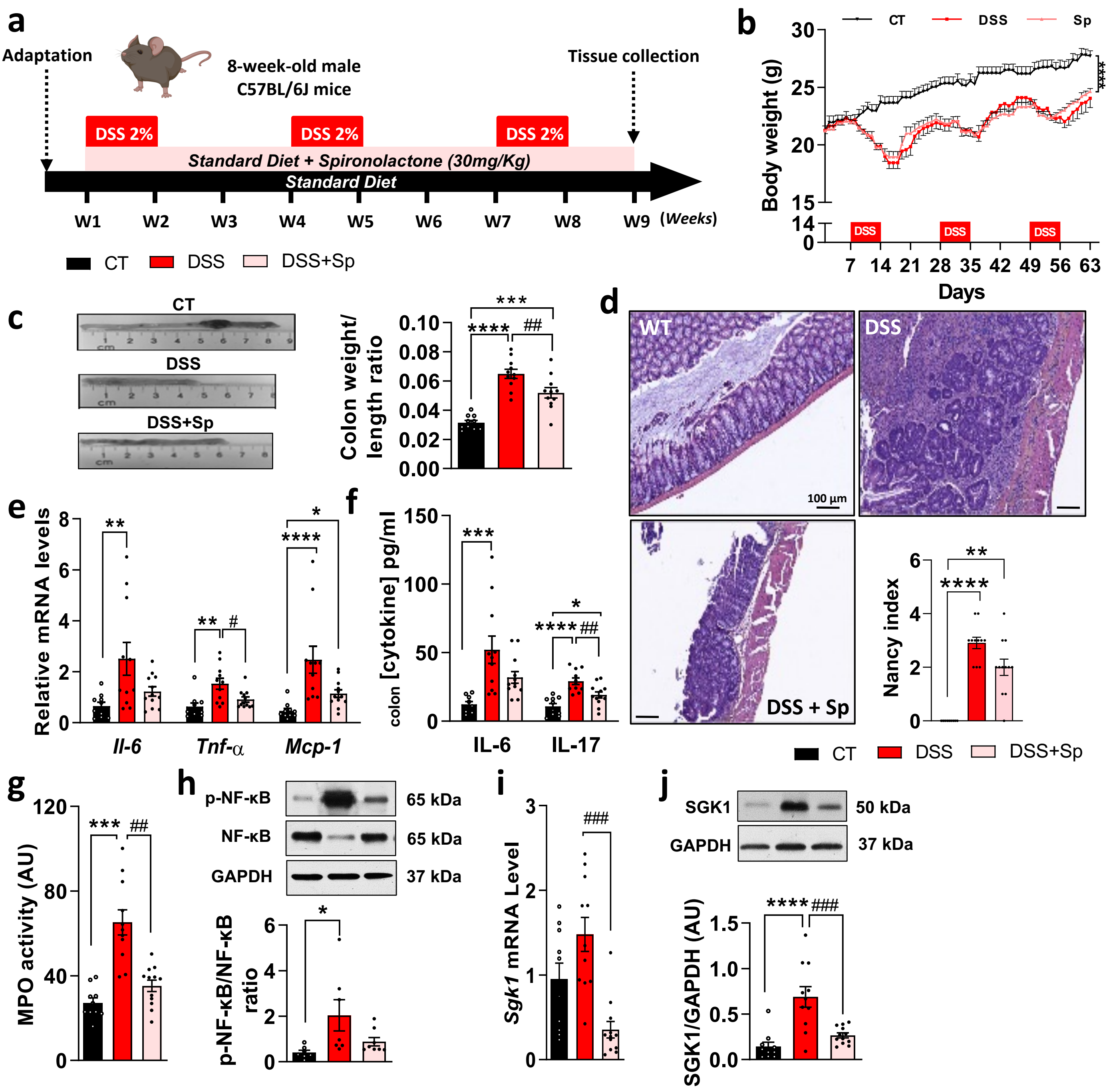

Figure S1. Pharmacological inhibition of mineralocorticoid receptor (MR) by spironolactone (Sp) decreases intestinal inflammation in mice with dextran sulfate sodium (DSS)-induced chronic colitis. Male C57BL/6 mice underwent 3 cycles of 2% DSS in their drinking water for 7 days, followed by 14 days of regular water (DSS; n=11) for both first cycles and 7 days for the third cycle. Control mice received normal water (CT; n=10). Mice were subjected to either a standard diet or diet supplemented with spironolactone (30mg.kg^{-1}; DSS+Sp, n=12) throughout the experiment. (**a**) Experimental design. (**b**) Body weight (n=10 for CT, n=12 for DSS, n=12 for DSS+Sp). (**c**) Representative image of mice colon and colon weight/length ratio (n=10 for CT, n=11 for DSS, n=11 for DSS+Sp). (**d**) Hematoxylin-eosin-safran stained tissues in CT, DSS and DSS+Sp groups and Nancy score (n=9 for CT, n=11 for DSS, n=12 for DSS+Sp). (**e**) Relative colon mRNA level encoding for Il-6, Tnf-α and Mcp-1 (n=10 for CT, n=11 for DSS, n=12 for DSS+Sp). (**f**) Colon IL-6 and IL-17 levels (n=10 for CT, n=11 for DSS, n=12 for DSS+Sp). (**g**) Colon MPO activity (n=10 for CT, n=11 for DSS, n=12 for DSS+Sp). (**h**) Relative protein expression of phospho-NF-κB (p-NF-κB) and NF-κB and representative image of western blot (n=7 for CT, n=7 for DSS, n=8 for DSS+Sp). (**i**) Colonic relative mRNA and (**j**) protein expression level of MR target: SGK1 and representative image of SGK1 (n=10 for CT, n=11 for DSS, n=12 for DSS+Sp). Data are presented as mean values +/- SEM. Created in BioRender. Marion-letellier, R. (2025) https://BioRender.com/ lmfrrrn .

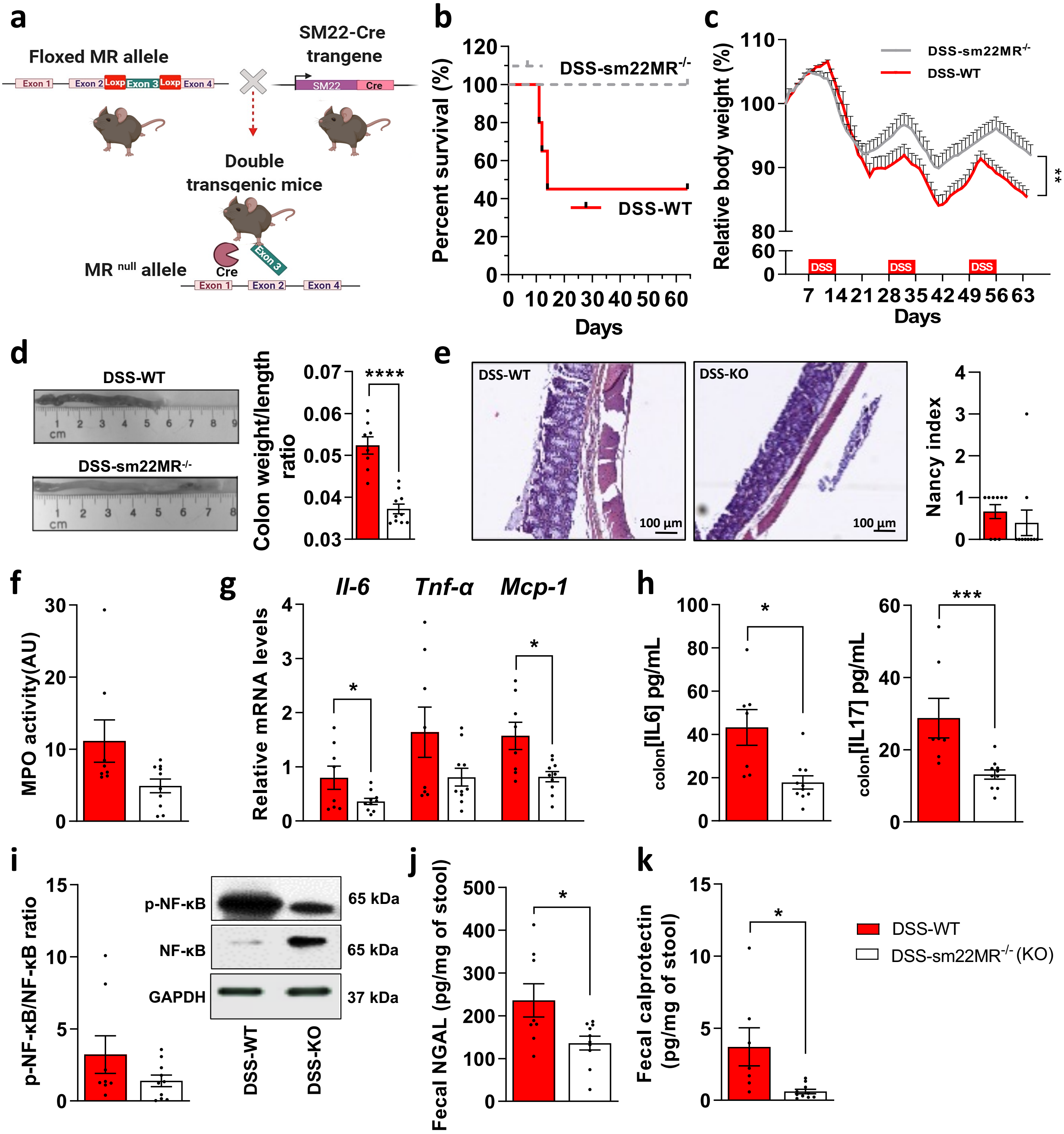

Figure S2: Genetic smooth muscle deletion of mineralocorticoid receptor (MR) decreases intestinal inflammation in female mice with chronic colitis.

Female C57BL/6J mice underwent 3 cycles of 2% DSS in their drinking water for 7 days, followed by 14 days of regular water for the both first cycles and 7 days for the third cycle (DSS-WT, n=8; DSS-sm22MR$^{-/-}$, n=10). (**a**) Transgenic animal design. (**b**) Survival curve. (**c**) Body weight (n=8 for DSS-WT, n=10 for DSS-sm22MR$^{-/-}$). (**d**) Representative image of mice colon and colon weight/length ratio (n=8 for DSS-WT, n=10 for DSS-sm22MR$^{-/-}$). (**e**) Hematoxylin-eosin-safran stained tissues in DSS-WT and DSS-sm22MR$^{-/-}$ groups and Nancy score (n=8 for DSS-WT, n=10 for DSS-sm22MR$^{-/-}$). (**f**) Colonic MPO activity (n=8 for DSS-WT, n=10 for DSS-sm22MR$^{-/-}$). (**g**) Relative colon mRNA level encoding for Il-6, Tnf-α and Mcp-1 (n=8 for DSS-WT, n=10 for DSS-sm22MR$^{-/-}$). (**h**) Colonic IL-6 and IL-17 levels (n=7 for DSS-WT, n=10 for DSS-sm22MR$^{-/-}$). (**i**) Representative western blot and relative protein expression of phospho NF-κB (p-NF-κB) and NF-κB (n=8 for DSS-WT, n=10 for DSS-sm22MR$^{-/-}$). (**j**) Fecal NGAL (n=8 for DSS-WT, n=10 for DSS-sm22MR$^{-/-}$) and (**k**) calprotectin (n=7 for DSS-WT, n=10 for DSS-sm22MR$^{-/-}$). Data are presented as mean values +/- SEM. Created in BioRender. Marion-letellier, R. (2025) https://BioRender.com/ lmfrrrn .

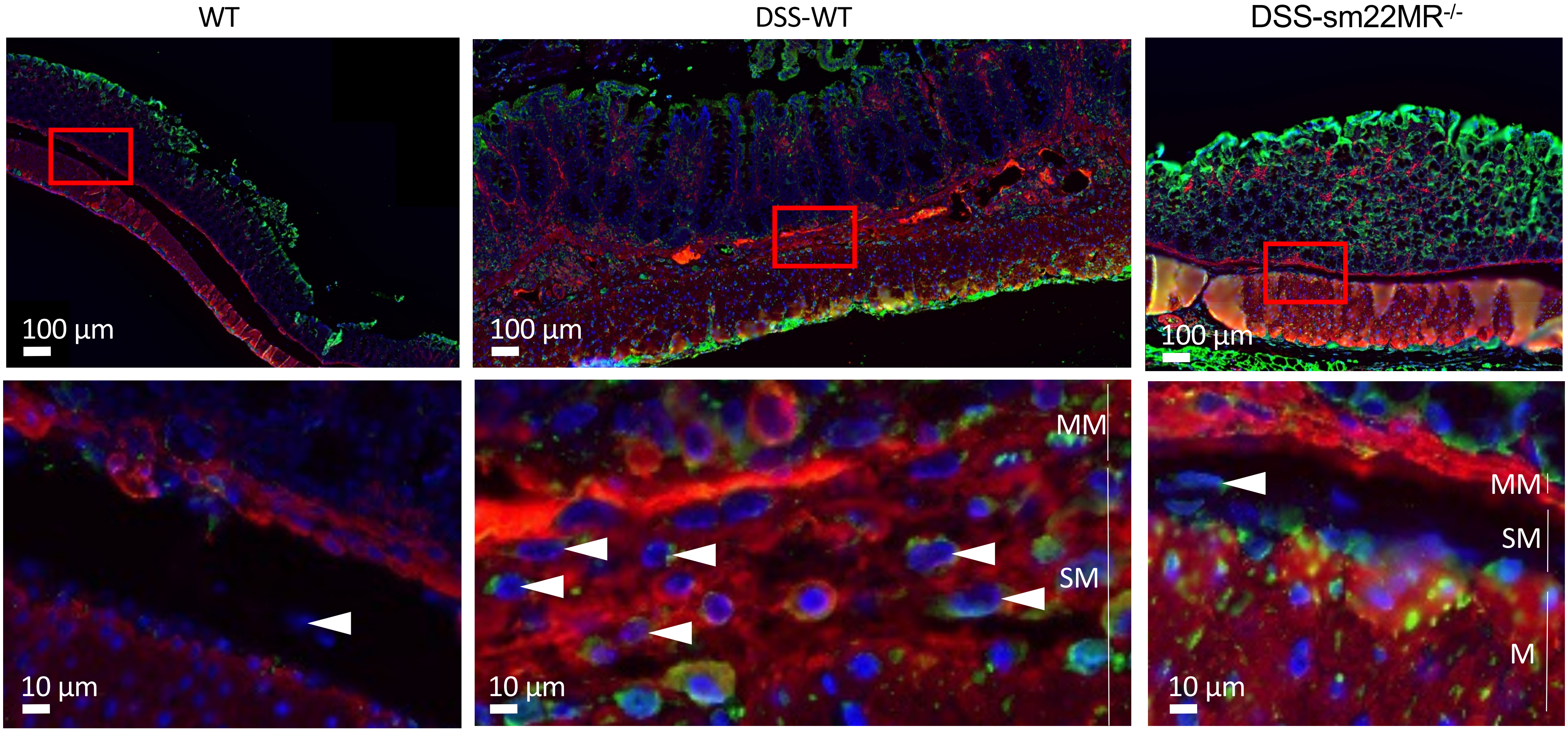

Figure S3 : Representative images of immunofluorescence double staining of NR3C2 (green) and SMA (red) of colon sections from WT (top line), DSS-WT (middle line) and DSS-sm22MR$^{-/-}$ (KO, bottom line) mice. The second row represents a high magnification of the red rectangle zone. White triangles represent spindle cells with heterogenous low expression of SMA corresponding to myofibroblasts. MM=muscular mucosa ; SM=submucosa ; ML=muscular layer. Nucleus are stained with DAPI (blue). Brightness and contrast were increased for illustration purposes.

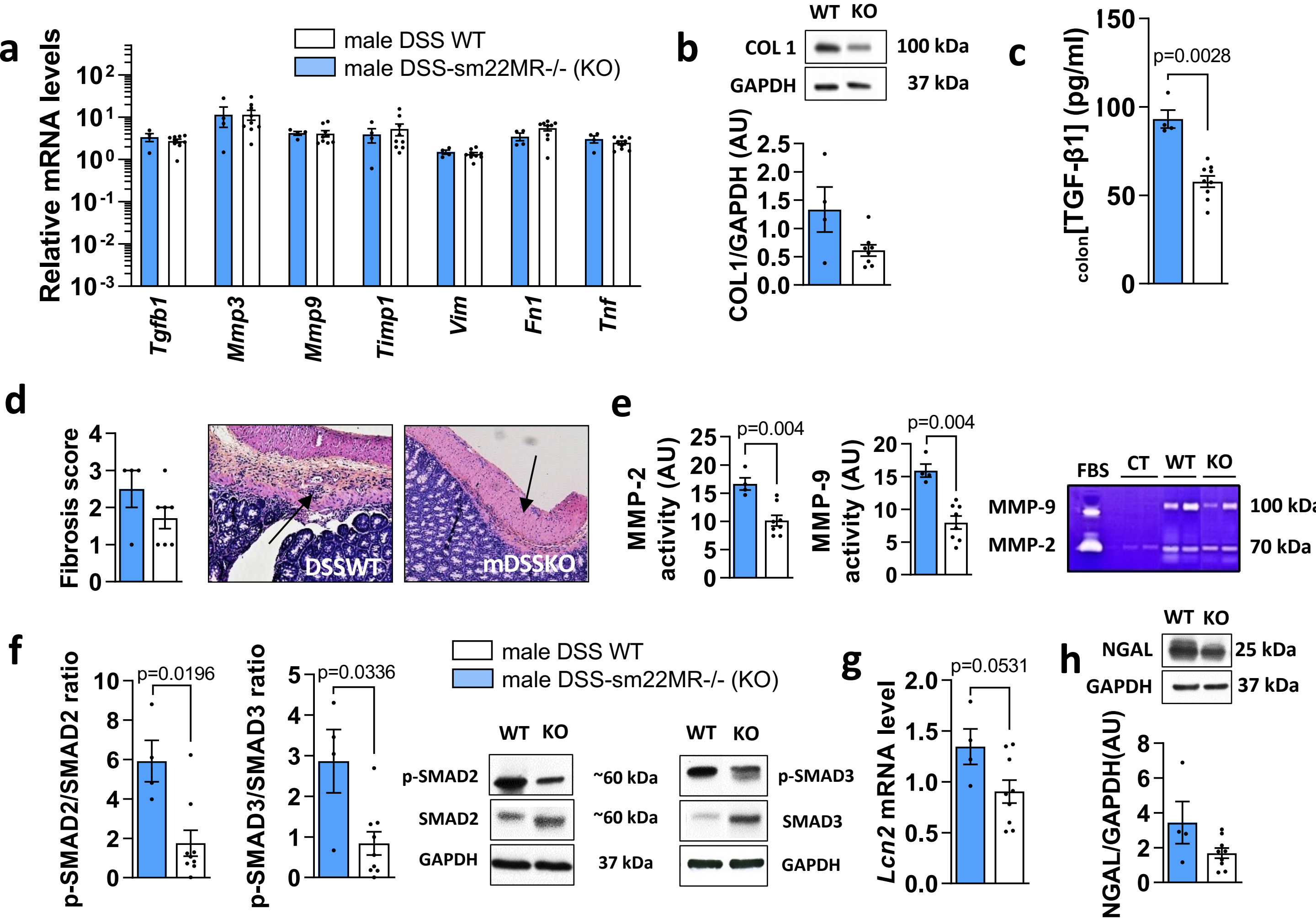

Figure S4: Genetic smooth muscle deletion of mineralocorticoid receptor (MR) decreases intestinal fibrosis in male mice with chronic colitis.

Male C57BL/6J mice underwent 3 cycles of 2% dextran sulfate sodium (DSS) in their drinking water for 7 days, followed by 14 days of regular water for the both first cycles and 7 days for the third cycle (DSS-WT, n=4; DSS-sm22MR$^{-/-}$, n=9).

(**a**) Colonic relative mRNA levels encoding for *Tgfb1*, *Mmp3*, *Mmp9*, *Timp1*, *Vim*, *Fn1* and *Tnf* (n=4 for DSS-WT, n=9 for DSS-sm22MR$^{-/-}$; Two-sided Mann-Whitney test). (**b**) Representative western blot and relative protein expression of COL1 (n=4 for DSS-WT, n=8 for DSS-sm22MR$^{-/-}$; Two-sided Mann-Whitney test). (**c**) Colonic TGF-β1 level (n=4 for DSS-WT, n=9 for DSS-sm22MR$^{-/-}$; Two-sided Mann-Whitney test). (**d**) Hematoxylin-eosin-safran stained tissues and fibrosis score (n=4 for DSS-WT, n=7 for DSS-sm22MR$^{-/-}$; Two-sided Mann-Whitney test). (**e**) Representative gelatine zymography and quantification of colon MMP-9 and MMP-2 activity (n=4 for DSS-WT, n=8 for DSS-sm22MR$^{-/-}$; Two-sided Mann-Whitney test). (**f**) Representative western blot and relative protein expression of phospho-SMAD 2/3 (p-SMAD2, p-SMAD3) and SMAD 2/3 (n=4 for DSS-WT, n=9 for DSS-sm22MR$^{-/-}$; Two-sided Mann-Whitney test). **g-h**) Colonic relative mRNA expression (**g**) and protein level (**h**) of NGAL (n=4 for DSS-WT, n=9 for DSS-sm22MR$^{-/-}$; Two-sided Mann-Whitney test). Data are presented as mean values +/- SEM.

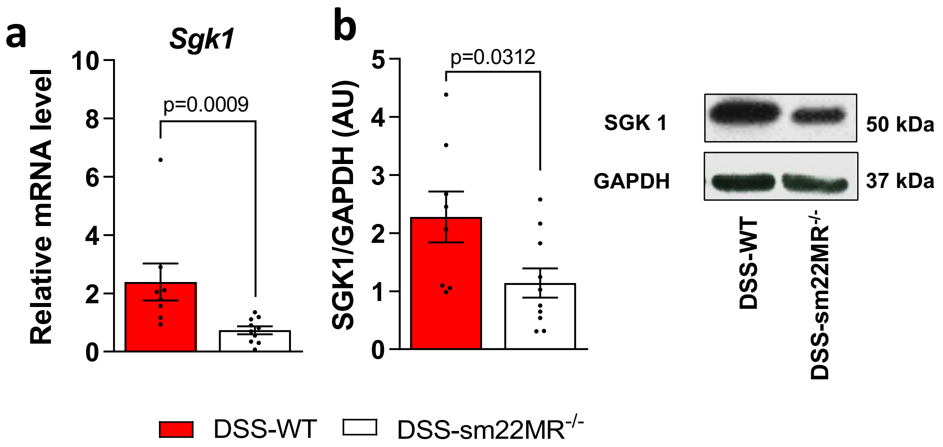

Figure S5: Colon SGK1 expression in male mice with genetic smooth muscle deletion of MR.
Male C57BL/6J mice underwent 3 cycles of 2% dextran sulfate sodium (DSS) in their drinking water for 7 days, followed by 14 days of regular water for the both first cycles and 7 days for the third cycle (DSS-WT, n=8; DSS-sm22MR$^{-/-}$, n=10). (**a**) Colonic relative mRNA levels encoding for *Sgk1* (n=8 for DSS-WT, n=10 for DSS-sm22MR$^{-/-}$; Two-sided Mann-Whitney test). (**b**) Colon protein expression of SGK1 and representative image of western blot (n=8 for DSS-WT, n=10 for DSS-sm22MR$^{-/-}$; Two-sided unpaired t test). Data are presented as mean values +/- SEM.

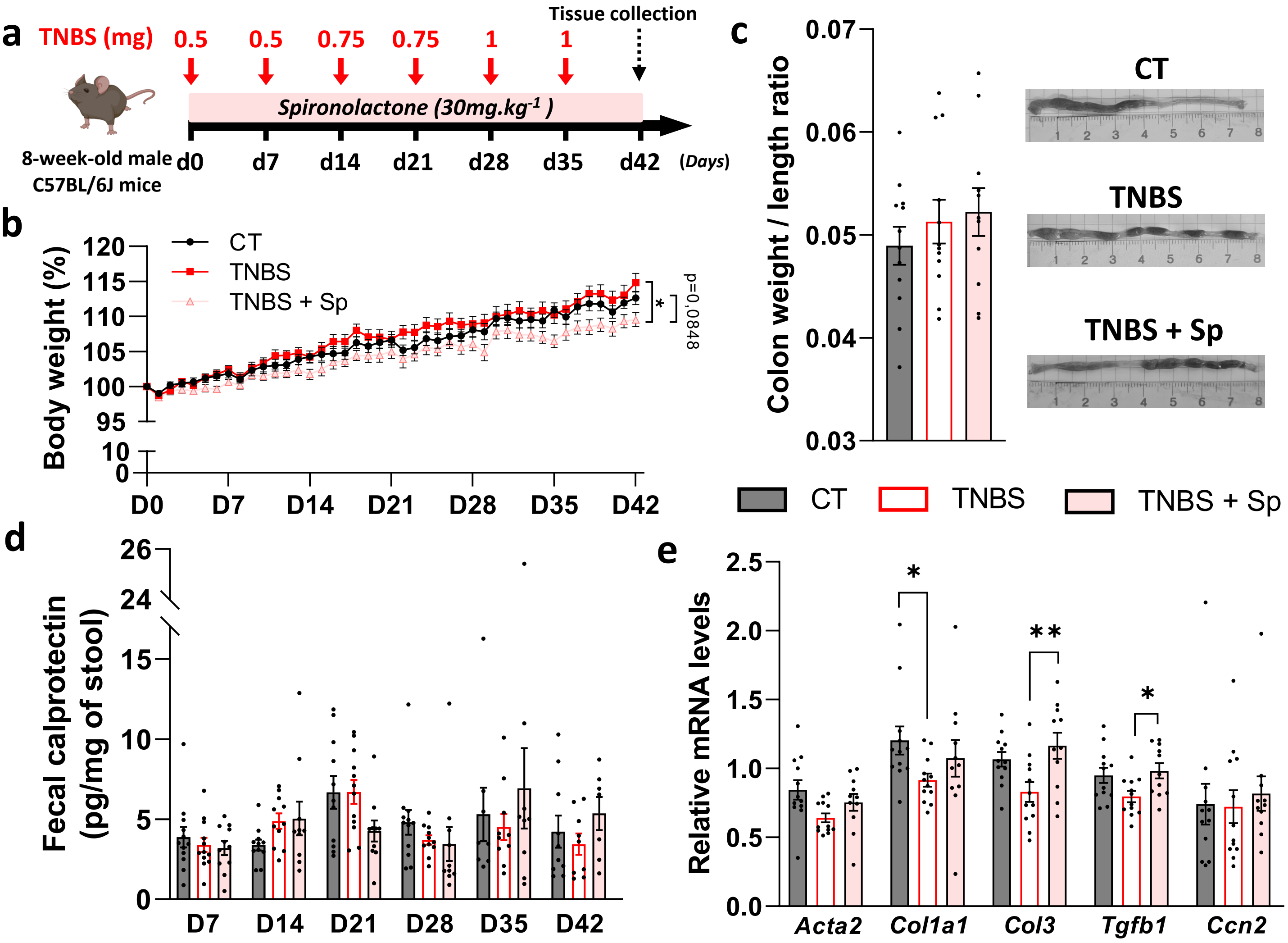

Figure S6: Pharmacological inhibition of mineralocorticoid receptor (MR) by spironolactone in male mice with trinitrobenzene sulfonic acid (TNBS)-induced chronic colitis.

Male C57BL/6 mice weekly underwent intrarectal injections of increasing concentration of TNBS (TNBS, n=12). Control mice received the vehicle (CT, n=12). Mice were subjected to either a standard diet or diet supplemented with spironolactone (30mg.kg^{-1}) throughout the experiment (TNBS + Sp, n=12). (**a**) Experimental design. (**b**) Body weight (n=12 per group). (**c**) Representative image of mice colon and colon weight/length ratio (n=12 for CT, n=12 for TNBS, n=11 for TNBS + Sp). (**d**) Fecal calprotectin (D7, n=12 for CT, n=12 for TNBS, n=11 for TNBS + Sp; D14, n=11 for CT, n=11 for TNBS, n=10 for TNBS + Sp; D21, n=11 for CT, n=12 for TNBS, n=10 for TNBS + Sp; D28, n=12 for CT, n=11 for TNBS, n=10 for TNBS + Sp; D35, n=8 for CT, n=10 for TNBS, n=9 for TNBS + Sp; D42, n=10 for CT, n=9 for TNBS, n=7 for TNBS + Sp). (**e**) Colonic relative mRNA levels encoding for *Acta2, Col1a1, Col3, Tgfb1* and *Ccn2* (n=12 for CT, n=12 for TNBS, n=11 for TNBS + Sp). Data are presented as mean values +/- SEM. Created in BioRender. Marion-letellier, R. (2025) https://BioRender.com/ lmfrrrn .

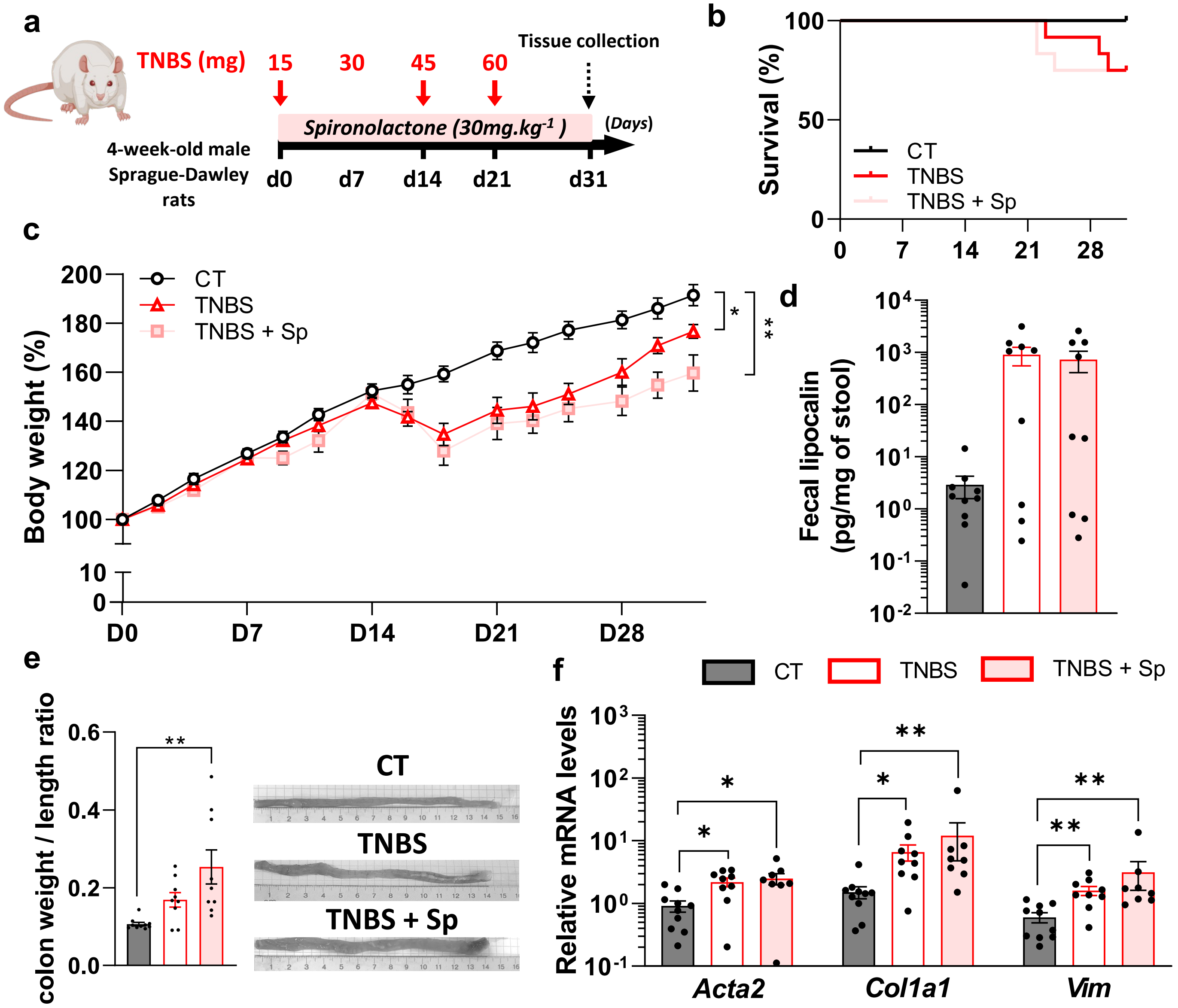

Figure S7: Pharmacological inhibition of mineralocorticoid receptor (MR) by spironolactone in male rats with trinitrobenzene sulfonic acid (TNBS)-induced chronic colitis.
Male Sprague-Dawley rats weekly underwent intrarectal injections of increasing concentration of TNBS for 4 weeks (TNBS, n=9). Control rats received the vehicle (CT, n=10). Rats were subjected to either a standard diet or diet supplemented with spironolactone (30mg.kg^{-1}) throughout the experiment (TNBS + Sp, n=9). (**a**) Experimental design. (**b**) Survival. (**c**) Body weight (n=10 for CT, n=9 for TNBS, n=9 for TNBS + Sp). (**d**) Fecal calprotectin (n=10 for CT, n=9 for TNBS, n=9 for TNBS + Sp). (**e**) Representative image of rat colon and colon weight/length ratio (n=10 for CT, n=9 for TNBS, n=9 for TNBS + Sp). (**f**) Colonic relative mRNA levels encoding for *Acta2, Col1a1* and *Vim* (n=10 for CT, n=9 for TNBS, n=8 for TNBS + Sp). Data are presented as mean values +/- SEM. Created in BioRender. Marion-letellier, R. (2025) https://BioRender.com/ lmfrrrn .

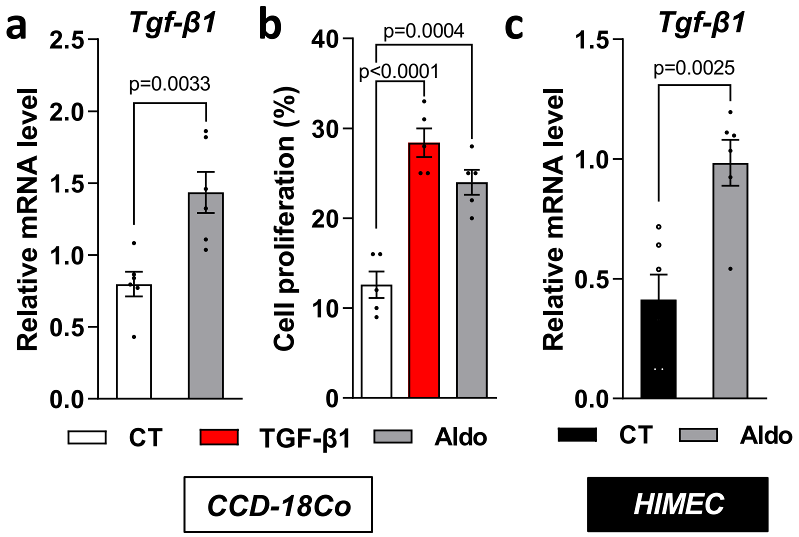

Figure S8: Effects of mineralocorticoid receptor activation on CCD-18Co and HIMEC cells.
CCD-18Co (**a,b**) and HIMEC (**c**) cells were incubated with aldosterone (100nM) or TGF-β (10 ng/mL) for 24h. (n=6 from independent experiments). (**a**) Relative mRNA level encoding for TGF-β1 from CCD18-co cell lysates (n=6 per group; Two-sided unpaired t test). (**b**) CCD-18Co cells proliferation (n=5 per group, ordinary one-way ANOVA with Tukey's multiple comparisons test). (**c**) Relative mRNA encoding for TGF-β1 from HIMEC cell lysates (n=6 per group; Two-sided unpaired t test). Data are presented as mean values +/- SEM.

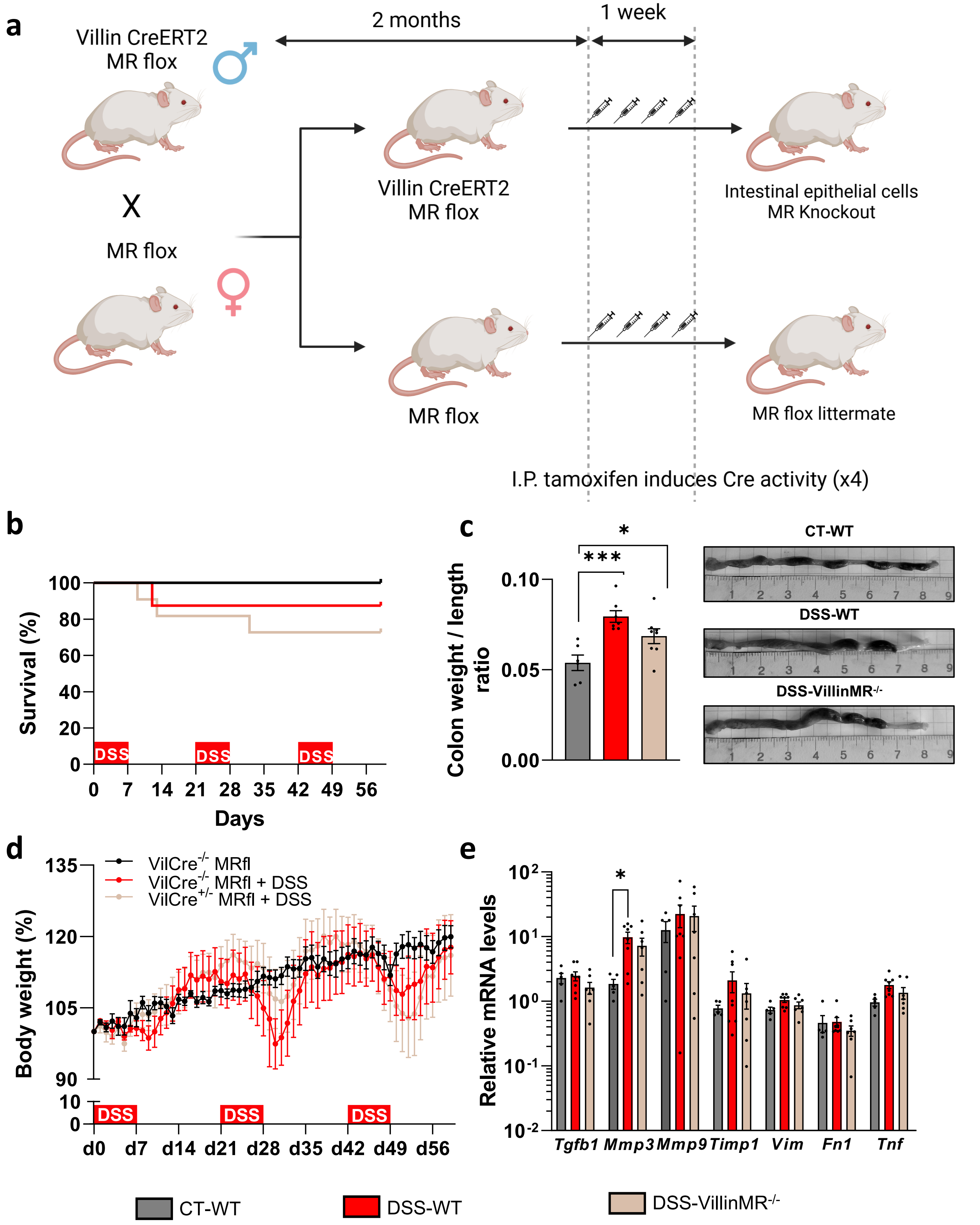

Figure S9: Genetic intestinal epithelial deletion of mineralocorticoid receptor (MR) did not impact inflammation or intestinal fibrosis in male mice with chronic colitis.

Male C57BL/6J mice underwent 3 cycles of 1% DSS in their drinking water for 7 days, followed by 14 days of regular water for the both first cycles and 7 days for the third cycle (DSS-WT, n=7; DSS-VillinMR$^{-/-}$, n=8). Control mice received normal water (CT-WT, n=6). (**a**) Transgenic animal design. (**b**) Survival rate. (**c**) Representative image of mice colon and colon weight/length ratio (n=6 for CT-WT, n=7 for DSS-WT, n=8 for DSS-VillinMR$^{-/-}$). (**d**) Body weight (n=6 for CT-WT, n=7 for DSS-WT, n=8 for DSS-VillinMR$^{-/-}$). (**e**) Colonic relative mRNA levels encoding for *Tgfb1*, *Smad2*, *Smad3*, *Mmp3*, *Mmp9*, *Timp1* and *Tnf* (n=5 for CT-WT, n=8 for DSS-WT, n=7 for DSS-VillinMR$^{-/-}$). Data are presented as mean values +/- SEM. Created in BioRender. Marion-letellier, R. (2025) https://BioRender.com/ lmfrrrn.

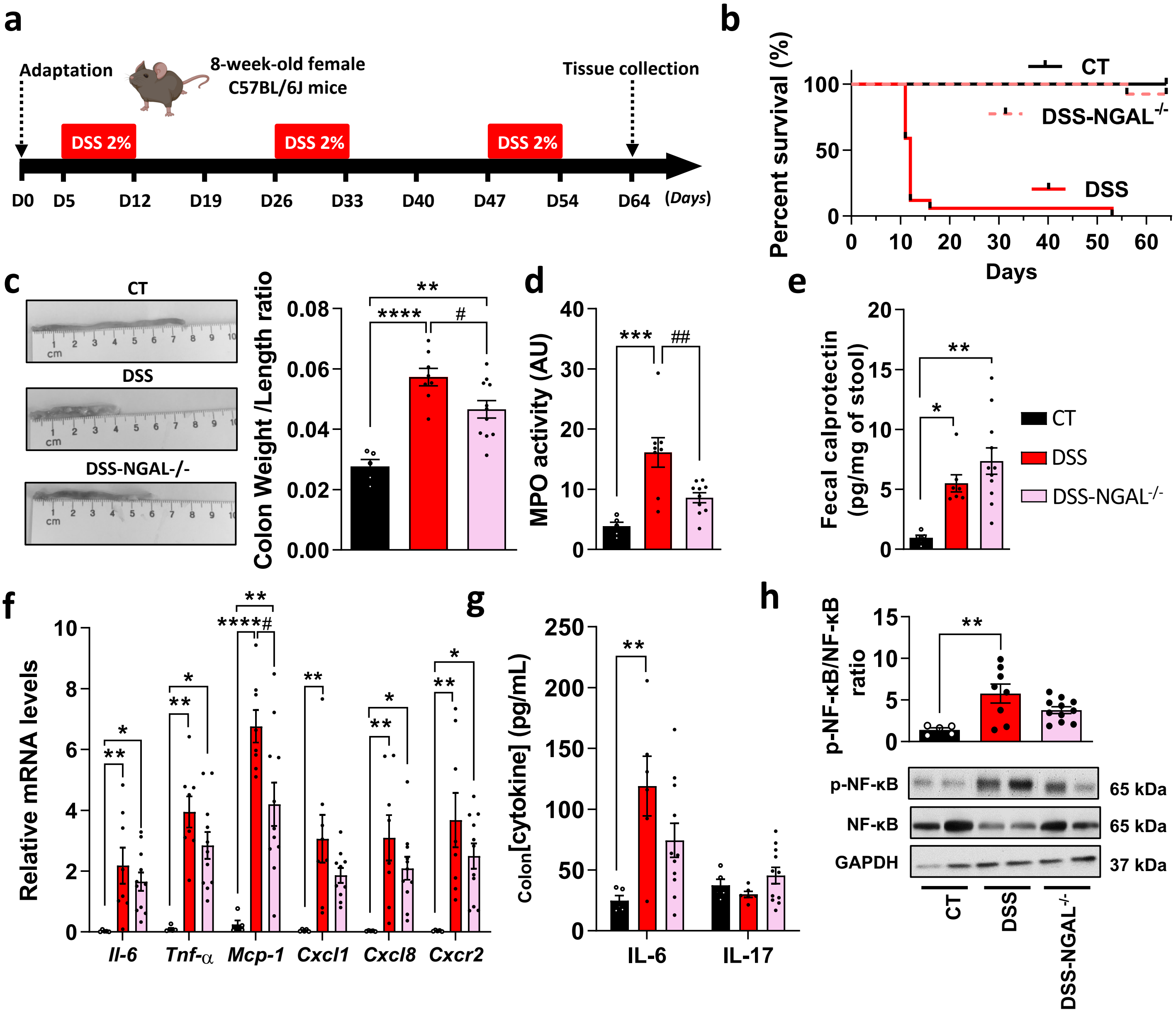

Figure S10: Effect of genetic deletion of neutrophil gelatinase-associated lipocalin (NGAL) on intestinal inflammation in female mice with chronic dextran sulfate sodium (DSS)-induced colitis.

Female C57BL/6 DSS-NGAL$^{-/-}$ (n=13) and DSS (n=8) underwent 3 cycles of 2% DSS in their drinking water for 7 days, followed by 14 days of regular water for both first cycles and 7 days for the third cycle. Control mice received normal water (CT; n=10). (**a**) Experimental design. (**b**) Survival curve. (**c**) Representative image of mice colon and colon weight/length ratio (n=5 for CT, n=8 for DSS, n=11 for DSS-NGAL$^{-/-}$). (**d**) Colonic MPO activity (n=5 for CT, n=8 for DSS, n=10 for DSS-NGAL$^{-/-}$). (**e**) Fecal calprotectin (n=5 for CT, n=7 for DSS, n=11 for DSS-NGAL$^{-/-}$). (**f**) Relative colon mRNA level expression of Il-6, Tnf-α, Mcp-1, Cxcl1, Cxcl8 and Cxcr2 (n=5 for CT, n=8 for DSS, n=11 for DSS-NGAL$^{-/-}$). (**g**) Colon IL-6 and IL-17 levels (n=5 for CT, n=6 for DSS, n=11 for DSS-NGAL$^{-/-}$). (**h**) Colonic phosphorylation of NF-κB (n=5 for CT, n=8 for DSS, n=11 for DSS-NGAL$^{-/-}$). Data are presented as mean values +/- SEM. Created in BioRender. Marion-letellier, R. (2025) https://BioRender.com/lmfrrrn.

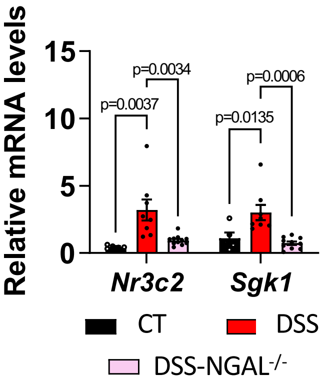

Figure S11: Colonic mineralocorticoid receptor (MR) pathway in chronic dextran sulfate sodium (DSS) mice deleted for NGAL.
Female C57BL/6 DSS-NGAL$^{-/-}$ (n=13) and DSS (n=8) underwent 3 cycles of 2% DSS in their drinking water for 7 days, followed by 14 days of regular whereas for both first cycles and 7 days for the third cycle. Control mice received normal water (CT-WT; n=10). Colonic relative mRNA levels encoding for MR (Nr3c2) and MR target: Sgk1 (*Nr3c2*, n=5 for CT, n=8 for DSS, n=11 for DSS-NGAL$^{-/-}$; Sgk1, n=5 for CT, n=8 for DSS, n=10 for DSS-NGAL$^{-/-}$; ordinary one-way ANOVA with Tukey's multiple comparisons test). Data are presented as mean values +/- SEM.

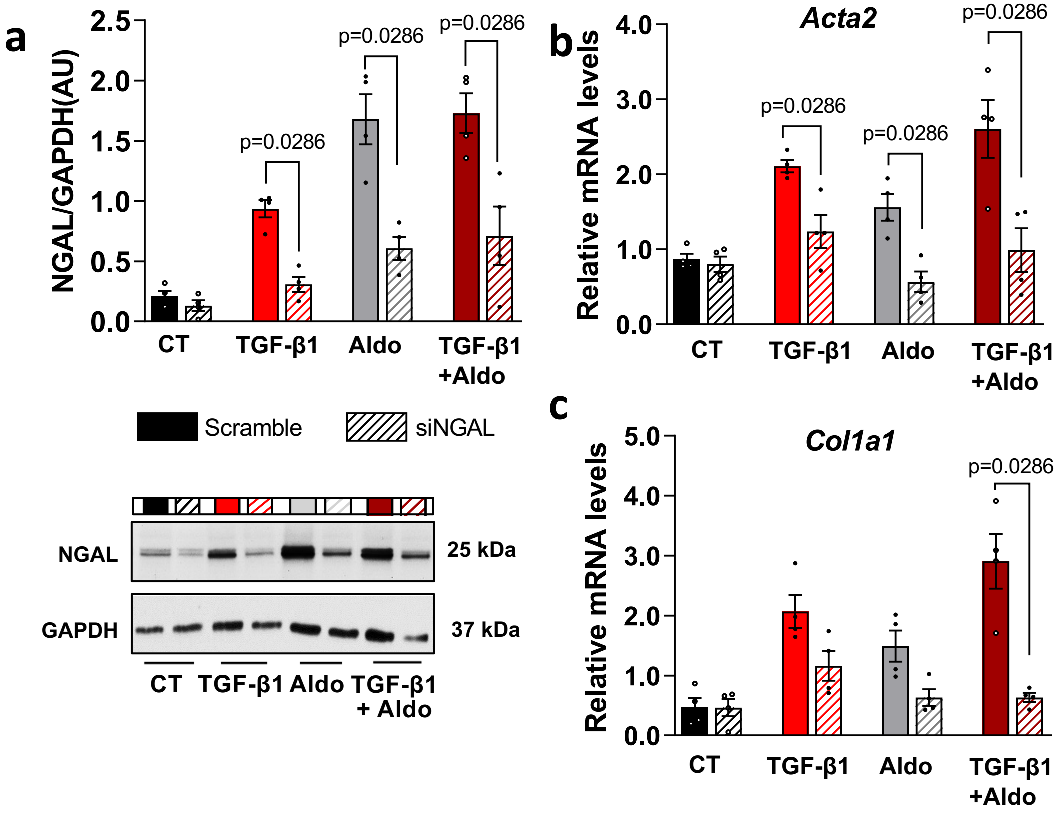

Figure S12: Effects of neutrophil gelatinase-associated lipocalin (NGAL) silencing in CCD-18Co fibroblasts.

CCD-18Co cells silenced for NGAL with siNGAL, were incubated with aldosterone (Aldo, 100nM) with or without TGF-β (10ng/mL) for 24h. (n=4 from independent experiments). (**a**) Representative western blot and relative protein expression of NGAL (n=4 per group; Two-sided Mann-Whitney test). (**b-c**) Relative mRNA level encoding for α-sma (**b**) and Col1a1 (**c**) from CCD-18Co cells lysate (n=4 per group; Two-sided Mann-Whitney test). Data are presented as mean values +/- SEM.

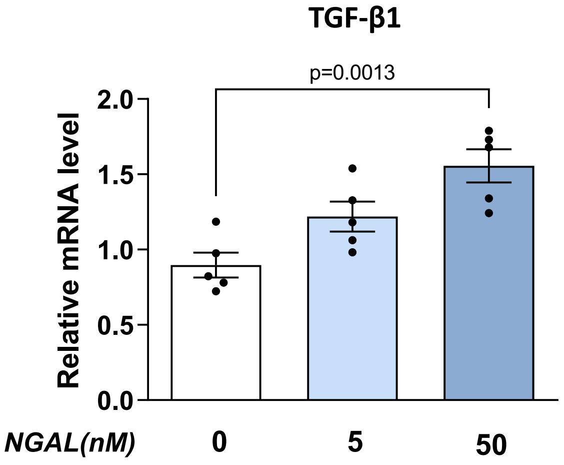

Figure S13: Effects of neutrophil gelatinase-associated lipocalin (NGAL) in CCD-18Co fibroblasts. CCD-18Co cells were incubated with increasing concentration of recombinant hNGAL (5ng/ml and 50ng/ml; n=5 from independent experiments). Relative mRNA level of TGF-β1 from CCD-18Co cells lysates (n=5 per group; ordinary one-way ANOVA with Tukey's multiple comparisons test). Data are presented as mean values +/- SEM.

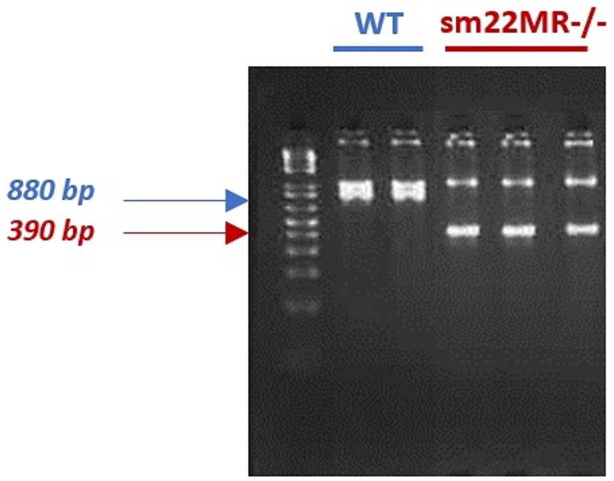

Figure S14: Example of PCR of SM22MR-/- mice genotyping.

Supplemental Table 1: Primer for genotyping

Mouse	Forward (5'-->3')	Reverse (5'-->3')
Sm22 Cre	GCA CAT GTT CAG CCA TCG CCA GGC G	GCA TAA CCA GTG AAA CAG CAT TGC TG
dMR	CTG GAG ATC TGA ACT CCA GGC T	CCT AGA GTT CCT GAG CTG CTG A
LoxP	CTG GAG ATC TGA ACT CCA GGC T	TAG AAA CAC TTC GTA AAG TAG AGC T

Supplemental Table 2: Primer sequences (PCR arrays)

Mouse	Forward (5'-->3')	Reverse (5'-->3')
Eef2	AAG CGG CTG GCT AAG TCT GA	GGC GCC AGC AAT AAT ATG TTC
Fn1	CCG GCT ACA TCA TCA AGT ATG AGA	TCT GAT TAT TCT TCA GGG CAA TGA C
Mmp3	CCC AGG AAG ATA GCT GAG GAC TT	GTA GAG AAA CCC AAA TGC TTC AAA G
Mmp9	CGT GTC TGG AGA TTC GAC TTG A	TGG TAC TGG AAG ATG TCG TGT GA
Rps18	GAA CAG ACA GAA GGA TGT GAA GGA	CCT CAC GCA GCT TGT TGT CTA G
Smad2	CCA TCA AAC TCG GAG AGG TTC T	ACT CCC CTT CCT ATA TGT CTT CTT GTC
Smad3	TGC AGC CGT GGA ACT TAC AAG	GAC CTC CCC TCC GAT GTA GTA GA
Tbp	TGC CCA GCA TCA CTA TTT CA	TTC TGG TCC ATG ATT CTC CC
Tgfb1	TGG AGC AAC ATG TGG AAC TC	CAG CAG CCG GTT ACC AAG
Timp1	GCA AAG AGC TTT CTC AAA GAC C	AGG GAT AGA TAA ACA GGG AAA CAC T
Tnf	CAC CGT CAG CCG ATT TGC	TGA GTT GGT CCC CCT TCT CC
Vim	GAG TCC ACT GAG TAC CGG AGA CA	GGG ACT CAT TGG TTC CTT TAA GG

Supplemental Table 3: Primer sequences (qPCR)

Mouse	Forward (5'-->3')	Reverse (5'-->3')	T of annealing (°C)
Acta2	AAG AGG AAG ACAG CAC AGC C	AGC GTC AGG ATC CCT CTC TT	60
Ccn2	TGA CCC CTG CGA CCC ACA	TAC ACC GAC CCA CCG AAG ACA CAG	68
Col1a1	CAG ACT GGC AAC CTC AAG AA	GGC CAA TGT CTA GTC CGA AT	60
Col3	AAC ACG AGG TCC TTC AGG TG	ATC CAT CTT TGC CAT CTT CG	60
Cxcl1	ACT CAA GAA TGG TCG CGA GG	GGG ACA CCT TTT AGC ATC TTT TGG	60
Cxcl8	GAT TCA CCT CAA GAA CAT CCA GA	GGA CAC CTT TTA GCA TCT TTT GG	60
Cxcr2	TGG CTG GGATTCACCTCAAGAACA	TGT GGC TAT GAC TTC GGT TTG GGT	60
Gapdh	CAT CAC TGC CAC CCA GAA GA	AAG TCG CAG GAG ACA ACC T	60
Il6	TAG TCC TTC CTA CCC CAA TTT CC	TTG GTC CTT AGC CAC TCC TTC	66
Lcn2	GGA CCA GGG CTG TCG CTA CT	GGT GGC CAC TTG CAC ATT GT	64
Mcp-1	TTA AAA ACC TGG ATC GGA ACC AA	GCA TTA GCT TCA GAT TTA CGG GT	65
Nr3c2	CTT TGG CAG TTT CCC AGT GC	CAC ATT GCT CGC ATG TAC GG	62
Sgk1	ATG CAG TAA ACC AAG CCG GT	CTT GAT CCA TCT TCG TAC CCG T	61
Tgfb1	CAC TCC CGT GGC TTC TAG TG	CTT CGA TGC GCT TCC GTT TC	64
Tnf	TGT CTA CTC CTC AGA GCC CC	TGA GTC CTT GAT GGT GGT GC	65
**Human**			
ACTA2	ACT GCC TTG GTG TGT GAC AA	CAC CAT CAC CCC CTG ATG TC	62
CCN2	TAG GAG CAG TGG GAG GGT AC	GGC TGG AGA ATG CAC ATC CT	63
COL1A1	CCA GAC CAG GAA TTC GGC TT	TGC TTG TCT GTT TCC GGG TT	63
COL3	CAC GGA AAC ACT GGT GGA CAG ATT	ATG CCA GCT GCA CAT CAA GGA C	70
CYP11B2	GCA CAA ATG TGG CGT GTT CT	CTT CTT CTT CAG GGC CTG GG	62
GAPDH	TGC CAT CAA TGA CCC CTT CA	TGA CCT TGC CCA CAG CCT TG	62
NR3C2	ACG GTG GGG TCA AGT TTC TC	TAG CAC CGG AAA CAC AGC TT	61
TGFB1	CAA GCA GAG TAC ACA CAG CAT	TGC TCC ACT TTT AAC TTG AGC C	63
**Rat**			
Acta2	CTT CTA TAA CGA GCT TCG C	TCC AGA GTC CAG CAC AAT	54
Actb	ACA ACC TTC TTG CAG CTC CTC	CTG ACC CAT ACC CAC CAT CAC	60
Col1	ACT CAG CCC TCT GTG CCT	CCT TCG CTT CCA TAC TCG	54

Eef2	TGT TCG TGG TCA AGG CCT AC	TCA GCT ACA ACT TGG CTG GG	60
Gapdh	CAT CAC TGC CAC TCA GAA GA	AAG TCA CAG GAG ACA ACC T	60
Vim	ATG AAA GTG TGG CTG CCA AGA AC	GTG ACT GCA CCT GTC TCC GGT A	55

Supplemental Table 4: Antibodies

Antibody	Working Dilution	Reference
α-SMA	1/5000	A5828, Merck
Collagen I	1/1000	Sc293182, Santa Cruz
MR	1/1000	MABS496, Millipore
p-SMAD2	1/1000	Ab63576, Abcam
p-SMAD3	1/1000	Ab52903, Abcam
Smad2	1/1000	Ab63576, Abcam
Smad3	1/1000	Ab28379, Abcam
NGAL	1/1000	Ab63929, Abcam
SGK1	1/1000	Sc28338, Santa Cruz
GAPDH	1/5000	SAB25500541, Merck

Supplemental method

VillinMR$^{-/-}$ transgenic mice.

Villin-Cre^{ERT2} MRfl male mice were kindly provided by Jaisser's lab (Cordeliers Research Center, INSERM U1138, Paris, France). C57BL/6JRj mice carrying a transgene with tamoxifen inducible Cre recombinase under the villin promoter were crossed with floxed MR mice. Specific invalidation of MR in intestinal epithelial cells was performed before each experiment by intraperitoneal injections of tamoxifen solution (1 mg, Merck).

2,4,6-trinitro-benzene sulfonic acid (TNBS)- induced chronic colitis.

TNBS-induced chronic colitis was performed in mice (8-week-old C57BL/6JRj male) and rats (4-week-old Sprague-Dawley male) purchased from Janvier Labs (Le Genest St Isle, France). Animals were divided into 3 groups of 12: Control (CTL) ; TNBS and TNBS + spironolactone (TNBS+ Sp). Water and food were provided ad libitum. TNBS was purchased from Merck and diluted in 30% of ethanol.

Chronic TNBS protocol was performed as previously described[21 32]. In brief, chronic colitis was induced by weekly intra-rectal instillation of increased concentration of TNBS ([0.5, 0.5, 0.75, 0.75, 1, 1mg] for mice and [15, 30, 45 and 60mg] for rats). Before the each TNBS instillation, a short fasting of 12h precedes. CTL groups received only vehicle solution (30% ethanol in PBS). Spironolactone was added to powder diet (SAFE, Augy, France) at 30mg.kg-1.

Euthanasia and sampling in mice study. Mice were killed by an overdose of anaesthesia (ketamine/ Xylazine solution, 40 and 1 mg. kg^{-1}, respectively). Blood was collected, after centrifugation (4°C, 3000 g, 15 min) plasma was collected and stored at -80°C. Colons were excised, measured weighed and immediately frozen in nitrogen before stored at -80°C until analysis.

Proliferation assay. CCD-18Co cells were seeded in Petri culture dishes at a density of 150,000 cells. After 24 h, cells were incubated with 10ng/mL of human recombinant TGF-β1 (PeproTech, USA), and/or aldosterone (100nM; Sigma-Aldrich, USA) for 24 hours. Cells were then detached using Trypsin-EDTA (Sigma-Aldrich, USA, T4049) and counted by Trypan Blue (Sigma-Aldrich, USA, T8154) exclusion to estimate the number of viable cells. Proliferation rate was calculated as the percentage of increased number of cells compared the number of seeded cells over 24 hours.

Mycoplasma analysis in cell study. Cell culture supernatant was collected. DNA extraction was performed using NucleoSpin® Tissue KIT according to the manufacturer's protocol (Macherey-Nagel, Germany) and specific Mycoplasma DNA was detected by qPCR using specific primer, Forward: 5' – GTTTGATCCTGGCTCAGGAYDAAC – 3' and Reverse: 5' – GAAAGGAGGTRWTCCAYCCSCAC – 3'.

Gene expression by quantitative RT-qPCR analysis. Mouse colon samples were homogenized in 1 ml of Trizol reagent (Invitrogen, USA) with the use of TissueLyser LT machine (Qiagen, UK) while cells were lysed in 500µL of Trizol reagent (Invitrogen, USA). 200µl and 100 µl of chloroform (VWR, USA) were adding respectively for colon sample and cells samples before centrifugation (12000 g, 15 min, 4°C). The aqueous phase was collected and mix with 250µl of isopropanol (VWR, USA) and centrifuged (12000g, 15 min, 4°C). The pellet was washed with 75% ethanol (VWR, USA) before taken up in RNase and DNase free water (Invitrogen, USA). After DNase treatment (Promega, France), a reverse transcription of 1 µg total RNA was performed into cDNA by using 200 units of SuperScript™ II Reverse Transcriptase (LifeTechnologies, USA). Real time PCR was performed by using SYBR™ Green technology on BioRad CFX96 real time PCR system (BioRad Laboratories, France). Specific primer sequences of targeted mouse or human genes were displayed in the **supplemental table 3**. Gapdh was used as endogenous reference gene (ThermoFisher, USA).

Protein expression by Western Blot. Colon samples of were homogenized in 300 µl of buffer lysis (Hepes 20 mM, KCl 300 mM, MgCl2 3mM, DTT 1mM, EDTA 0.2 mM with 0.25% NP40, 1% of phosphatase inhibitor and 0,5% of protease inhibitor) by using TissueLyser LT machine (Qiagen, UK). Supernatant were collected after centrifugation (12000 g, 15 min, 4°C). In *in vitro* study cells were lysed in 200µl of Cellytic™ buffer (Sigma-Aldrich, USA) and centrifuged (20000 g, 20min, 4°C) for obtained protein samples. Protein concentration was determinate by Bradford assay. SDS-PAGE system was using to separate 20-30µg of proteins samples diluted in loading buffer (1 M tris (pH 6.8), 2% Sodium Dodecyl Sulfate (SDS; Sigma-Aldrich, USA), 20% glycerol (Sigma-Aldrich, USA) and 0.02% bromophenol blue) on 4-20% gradient polyacrylamide gel (Biorad, France). Proteins were transferred to a PVDF membrane by liquid transfer system (Biorad, France). Membranes were blocked for 1h at room temperature with 5% of non-fat dry milk in Tris-buffered saline (10 mmol/L Tris, pH 8; 150 mmol/L NaCl) and 0.05% Tween 20 (TBST). After 3 TBST washes of 5min, membranes were incubated overnight at 4°C in 5% of bovine serum albumin (Eurobio, France) in TBST with primary antibodies (**supplemental table 4**). After 3 TBST washes of 10 min each, membranes were incubated in appropriate secondary antibodies (1/5000, Dako, Denmark) for 1h at room temperature. Immunocomplexes were revealed by enhanced chemiluminescence detection (ECL) kit (Biorad, France). Proteins bands were scanned (ImageScanner III; GE Healthcare) and analysed.

Colonic myeloperoxidase assay. Briefly, 1ml of Hexadecyl Trimethyl Ammonium solution (5 mg of HTAB into 1L of phosphate buffer) was added by sample for 50mg tissue. Samples were homogenized by sonication (30 Hz, 10 sec). 3 freezing (-80°C) and heat up (37°C) cycles of 15 min were performed. After centrifugation (15000 g, 15 min, 4°C) supernatant were collected. 50µl of each sample were added in duplicate into a 96-well plate. A standard range with human purified MPO was used (Sigma-Aldrich, USA). 200µL of o-dianisidine dihydrochloride (ODHC, 282 µg / mL; Sigma Aldrich, USA) mixture containing H_2O_2 (0.209 µg / mL; Sigma Aldrich, USA) was added. Measure absorbance at 450 nm using

a spectrophotometer was performed at 1, 2, 3, 5, 10, 15 and 20min. For analysis MPO activity protein levels were determined using Pierce ™ BCA Protein Assay Kit (Thermo Fisher Scientific, USA).
