# Supplementary material for: Mineralocorticoid receptor activation contributes to intestinal fibrosis through neutrophil gelatinase-associated lipocalin in preclinical models
Source: Nat Commun. 2025 Jul 9;16:6318. doi: 10.1038/s41467-025-61401-0 (PMC12241341; doi:10.1038/s41467-025-61401-0)
Supplement: Supplementary file 2 — Reporting Summary [file 41467_2025_61401_MOESM2_ESM.pdf]

nature portfolio

Corresponding author(s): Rachel Marion-Letellier

Last updated by author(s): 27th March 2025

Reporting Summary

Nature Portfolio wishes to improve the reproducibility of the work that we publish. This form provides structure for consistency and transparency in reporting. For further information on Nature Portfolio policies, see our Editorial Policies and the Editorial Policy Checklist.

Statistics

For all statistical analyses, confirm that the following items are present in the figure legend, table legend, main text, or Methods section.

n/a	Confirmed	
☐	☒	The exact sample size (*n*) for each experimental group/condition, given as a discrete number and unit of measurement
☐	☒	A statement on whether measurements were taken from distinct samples or whether the same sample was measured repeatedly
☐	☒	The statistical test(s) used AND whether they are one- or two-sided<br>*Only common tests should be described solely by name; describe more complex techniques in the Methods section.*
☐	☒	A description of all covariates tested
☐	☒	A description of any assumptions or corrections, such as tests of normality and adjustment for multiple comparisons
☐	☒	A full description of the statistical parameters including central tendency (e.g. means) or other basic estimates (e.g. regression coefficient) AND variation (e.g. standard deviation) or associated estimates of uncertainty (e.g. confidence intervals)
☐	☒	For null hypothesis testing, the test statistic (e.g. *F*, *t*, *r*) with confidence intervals, effect sizes, degrees of freedom and *P* value noted<br>*Give P values as exact values whenever suitable.*
☒	☐	For Bayesian analysis, information on the choice of priors and Markov chain Monte Carlo settings
☒	☐	For hierarchical and complex designs, identification of the appropriate level for tests and full reporting of outcomes
☐	☒	Estimates of effect sizes (e.g. Cohen's *d*, Pearson's *r*), indicating how they were calculated

Our web collection on statistics for biologists contains articles on many of the points above.

Software and code

Policy information about availability of computer code

Data collection	not applicable
Data analysis	not applicable

For manuscripts utilizing custom algorithms or software that are central to the research but not yet described in published literature, software must be made available to editors and reviewers. We strongly encourage code deposition in a community repository (e.g. GitHub). See the Nature Portfolio guidelines for submitting code & software for further information.

Data

Policy information about availability of data

All manuscripts must include a data availability statement. This statement should provide the following information, where applicable:

- Accession codes, unique identifiers, or web links for publicly available datasets
- A description of any restrictions on data availability
- For clinical datasets or third party data, please ensure that the statement adheres to our policy

We provide a file of our dataset through deposition in public repositories (Figshare).

Research involving human participants, their data, or biological material

Policy information about studies with human participants or human data. See also policy information about sex, gender (identity/presentation), and sexual orientation and race, ethnicity and racism.

Reporting on sex and gender	NA
Reporting on race, ethnicity, or other socially relevant groupings	NA
Population characteristics	NA
Recruitment	NA
Ethics oversight	NA

Note that full information on the approval of the study protocol must also be provided in the manuscript.

Field-specific reporting

Please select the one below that is the best fit for your research. If you are not sure, read the appropriate sections before making your selection.

☒ Life sciences ☐ Behavioural & social sciences ☐ Ecological, evolutionary & environmental sciences

For a reference copy of the document with all sections, see nature.com/documents/nr-reporting-summary-flat.pdf

Life sciences study design

All studies must disclose on these points even when the disclosure is negative.

Sample size	Sample size was determined by statistical approach taking account histological score as principal criteria (Tubbs et al., 2017).
Data exclusions	No data were excluded in the figures except some body weight gain data from the Fig.S8C because of an incident in the previous day compromising the value of the data.
Replication	The number of times cell experiments were independently replicated is reported in each figure legend.
Randomization	Mice and rats were randomly assigned to each experimental group according to their body weight.
Blinding	Our anatomopathologist was blinded to allocation and outcome assessment of experiments involving animal studies. For other experiments, blinding was not possible because data were largely analyzed by individual scientists who both collected and processed the samples.

Behavioural & social sciences study design

All studies must disclose on these points even when the disclosure is negative.

Study description	NA
Research sample	NA
Sampling strategy	NA
Data collection	NA
Timing	NA
Data exclusions	NA
Non-participation	NA
Randomization	NA

Ecological, evolutionary & environmental sciences study design

All studies must disclose on these points even when the disclosure is negative.

Study description	NA
Research sample	NA
Sampling strategy	NA
Data collection	NA
Timing and spatial scale	NA
Data exclusions	NA
Reproducibility	NA
Randomization	NA
Blinding	NA

Did the study involve field work? ☐ Yes ☒ No

Reporting for specific materials, systems and methods

We require information from authors about some types of materials, experimental systems and methods used in many studies. Here, indicate whether each material, system or method listed is relevant to your study. If you are not sure if a list item applies to your research, read the appropriate section before selecting a response.

Materials & experimental systems

n/a	Involved in the study	
☐	☒	Antibodies
☐	☒	Eukaryotic cell lines
☒	☐	Palaeontology and archaeology
☐	☒	Animals and other organisms
☒	☐	Clinical data
☒	☐	Dual use research of concern
☒	☐	Plants

Methods

n/a	Involved in the study	
☒	☐	ChIP-seq
☒	☐	Flow cytometry
☒	☐	MRI-based neuroimaging

Antibodies

Antibodies used	anti-α-SMA (A5828, Merck)<br>anti-COL1 (Sc293182, Santa Cruz)<br>anti-MR (MABS496, Millipore)<br>anti-pSMAD2 (Ab63576, Abcam)<br>anti-pSMAD3 (Ab52903, Abcam)<br>anti-SMAD2 (Ab63576, Abcam)<br>anti-NGAL (Ab63929, Abcam)<br>anti-SGK1 (Sc28338, SantaCruz)<br>anti-GAPDH (SAB25500541, Merck)
Validation	All antibodies employed in our manuscript were previously reported and routinely used for the application herein used.<br>All IHC antibodies were titrated on positive control tissue, starting from the recommended dilution. Respective staining without primary antibodies were used as a negative control.<br>All vendors used (Abcam, Merck, Santa Cruz Biotechnology and Millipore) report taking quality control measures to ensure that all antibodies sold are valid and reproducible. Validation information and citations for each antibody used herein could be found in the corresponding data sheets provided by the<br>commercial supplier.

Eukaryotic cell lines

Policy information about cell lines and Sex and Gender in Research

Cell line source(s)	CCD-18 Co, HIMEC and SMCs were obtained from American Type Culture Collection (ATCC), ScienCell Research Laboratories and CellBiologics, respectively.
Authentication	None of the cell line used were authenticated.
Mycoplasma contamination	CCD-18 Co, HIMEC and SMC were tested negative for mycoplasma contamination.
Commonly misidentified lines (See ICLAC register)	No commonly misidentified lines were used.

Animals and other research organisms

Policy information about studies involving animals; ARRIVE guidelines recommended for reporting animal research, and Sex and Gender in Research

Laboratory animals	Mice : C57BL/6J male and female between 8 and 9 weeks<br>Rats : 4 week-old Sprague-Dawley male
Wild animals	None of wild animal used.
Reporting on sex	Our data are diasaggregated for sex and we mentioned sex in the manuscript.
Field-collected samples	NA
Ethics oversight	All animals studies were conducted in accordance with the European directive for the use and care of laboratory animals (2010/63/UE) and received the agreement of the local animal ethics committee and of the ministerial committee for animal experimentation (#16184-2018071711054339).

Note that full information on the approval of the study protocol must also be provided in the manuscript.

Plants

Seed stocks	NA
Novel plant genotypes	NA
Authentication	NA
